# Occurrence and Strength of Instantaneous and Intracohort Density‐Dependence in Northeast Atlantic Fish Stocks

**DOI:** 10.1002/ece3.70375

**Published:** 2024-10-15

**Authors:** Massimiliano Cardinale, Valerio Bartolino, Henning Winker, Alessandro Orio, Christopher A. Griffiths, Laurie Kell

**Affiliations:** ^1^ Department of Aquatic Resources, Institute of Marine Research Swedish University of Agricultural Sciences Lysekil Sweden; ^2^ Centre for Environmental Policy Imperial College London London UK

**Keywords:** density‐dependence, intracohort analysis, reference points, spawning potential ratio, weight‐at‐age

## Abstract

Biological reference points (BRPs) used in fisheries management do not include density‐dependent (DD) growth, with DD processes only considered in the stock recruitment relationship. Not accounting for DD on somatic growth has led to criticism that such BRPs underestimate the compensatory effects of DD at low stock size, and therefore risk foregone catch opportunities. Here, we analyse 81 stocks from the Northeast Atlantic for evidence of DD growth, defined as the process in which stock size affects somatic weight. We evaluate the following questions: (1) How many stocks have experienced instantaneous DD growth and do stocks of the same species display similar trends? (2) Is there a common instantaneous DD growth relationship shared by all stocks? (3) For stocks exhibiting significant instantaneous DD growth, can we quantify the strength of the relationship? (4) Is DD growth operating as an intra‐cohort process as opposed to an instantaneous effect? Results reveal that only the weight of recruits exhibits a common instantaneous DD growth while the other responses analysed show a positive, noncompensatory effect, suggesting that other processes are at work. All responses examined showed significant temporal autocorrelation, which, when not accounted for, suggest apparent instantaneous DD growth in several stocks. Comparison of instantaneous against intracohort DD growth showed an increase in the number of stocks with significant DD growth, although, as for instantaneous DD growth, this declined greatly when temporal autocorrelation was accounted for. Our results counteract the a priori assumption that DD growth compensation is related only to stock biomass or density, suggesting that DD growth should be dealt case‐by‐case. Consequently, management practices that aim to fish down stock biomass with the anticipation of triggering DD growth will be associated with greater asymmetric risks than keeping biomass at levels where replacement yield does not rely on it.

## Graphical abstract

Around 80% of the tested key productivity parameters exhibit a positive global effect in somatic growth expressed as weight at age as opposed to the classic negative density‐dependent relationship when instantaneous growth is analysed. The frequency and strength of density‐dependence in weight at age is low and weak at the stock level when instantaneous growth is analysed. Significant temporal autocorrelation exists, which when not accounted for, reveals apparent density‐dependence in several stocks when instantaneous growth is analysed. The frequency of detection of density‐dependent weight at age increases when intracohort growth is analysed. However, even when considering intracohort growth, spurious density‐dependence in several stocks is detected when temporal autocorrelation is not accounted for, with at best 15% of the stocks exhibiting significant classic negative density‐dependence. Consequently, management practices that aim to fish down stock biomass with the anticipation of triggering density‐dependent growth, will be associated with greater asymmetric risks than keeping biomass at levels where replacement yield does not rely on it.

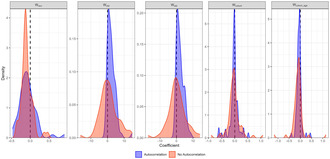

## 1 Introduction

Density‐dependence (DD) is a fundamental process in population ecology and is crucial for maintaining population stability and preventing populations from growing uncontrollably or declining to extinction (Turchin 1995). In population models of marine fishes, DD in recruitment is typically assumed to be the dominant regulatory population process (Rose et al. 2001; Thorson, Rudd, and Winker 2019; Zimmermann, Ricard, and Heino 2018). Theoretical and empirical evidence shows that DD can operate in numerous ways (Matthias et al. 2018), including competition for limited food resources, predation, cannibalism, spatial range contractions and evolutionary trait selection (Hixon, Pacala, and Sandin 2002; Bax 1998; Devine et al. 2012; Bartolino et al. 2011; Thorson et al. 2016). These processes can impact other key productivity traits, such as growth, survival and reproduction potential (Lorenzen and Enberg 2002; Devine et al. 2012; Zimmermann, Ricard, and Heino 2018; Perälä, Hutchings, and Kuparinen 2022). Consequently, an understanding of DD and its effects are particularly important for the management of exploited fish populations, because future catch advice will be intuitively sensitive to the assumptions made about the current and future productivity of a stock.

Population theory as well as several examples from the field and the laboratory predict that phenotypic DD will affect key productivity parameters (KPPs) such that growth, survival and maturation will be negatively related to population density (Engelhard and Heino 2004; Sparholt et al. 2021; Rindorf et al. 2022). At low density, reduced competition for limited resources results in higher density‐independent growth and survival rates, but, as the population increases towards carrying capacity, DD acts as a regulatory process reducing growth and survival to slow down the population growth rate. Increases in somatic growth at low abundance can also trigger earlier maturation because fish attain a size for maturation more quickly. The resulting hypothesis is that KPPs show compensatory responses at low density, associated with higher population growth rate, and therefore increased surplus production potential for fisheries (Sparholt et al. 2021). By contrast, depensatory DD is present if population growth is reduced at low densities, although empirical evidence is largely limited to depensatory DD in recruitment (e.g., Perälä and Kuparinen 2017; Perälä, Hutchings, and Kuparinen 2022).

In their analysis of DD and its effects on somatic growth, Rindorf et al. (2022) referred to the occurrence of positive relationships between their density measure and growth as ‘apparent positive DD’, which could be explained, for example, by an environmentally driven increase in food availability. Alternatively, a positive relationship between density and KPPs can also be a result of fishery‐induced evolution (Devine et al. 2012; Heino, Díaz Pauli, and Dieckmann 2015). An underlying hypothesis is that high fishing mortality can cause natural selection for early maturation (Heino, Díaz Pauli, and Dieckmann 2015; Claireaux, Jørgensen, and Enberg 2018). Furthermore, life history theory predicts that earlier investment into reproduction (e.g., gonadal development and mating) is negatively correlated with somatic growth and thus survival (Thorson et al. 2017; Kenchington 2014). There has also been experimental evidence of size‐selective harvesting being associated with smaller body sizes and earlier maturation in fish populations when compared to unharvested populations (Bouffet‐Halle et al. 2021). This is because in unharvested populations, density‐dependent natural selection favours larger body sizes at higher density (Bouffet‐Halle et al. 2021). The effects of density‐dependent natural selection, which favours larger body sizes at higher population densities, is conflicting with the effects of density‐dependent plasticity, which favours smaller body sizes at higher densities due to food limitation or social stress (Edeline and Loeuille 2020). Consistent with Rindorf et al. (2022), this paper focusses on DD in somatic growth (thereafter defined as DD growth) in a strictly compensatory sense, with no relationship, or a positive one, between a measure of density and the KPPs response, considered as evidence for no DD growth effects. That said, it is important to note that depensatory DD growth effects cannot be entirely ruled out as one of several nonexclusive mechanisms at play.

Modern fisheries management and advice are increasingly reliant on integrated stock assessment models due to their ability to deal with multiple datasets and complex data structures and their capacity to simultaneously fit to diverse data sets and estimate parameters related to biological and fishery processes (Maunder and Punt 2013). In several areas of the world (e.g., the USA and New Zealand) and in all tuna RFMOs (Regional Fisheries Management Organisations), several stocks are assessed using integrated stock assessment models and key reference points such as F_MSY_ (fishing mortality that achieves maximum sustainable yield), B_MSY_ (biomass at maximum sustainable yield) and B_0_ (biomass under unfished conditions) are directly derived from the model and used for management (Dichmont et al. 2016; Pons et al. 2016). In most cases, reference points rely on age‐structured time invariant assumptions (but see the concept of ‘dynamic B_0_’; Bessell‐Brown et al. 2022 and its practical application as to Northern shrimp *Pandalus borealis* (ICES 2022b)) and thus do not account for DD in KPPs. In Europe, ICES (International Council for the Exploration of the Sea) and GFCM (General Fisheries Commission for the Mediterranean), which provide advice for the Northeast Atlantic and the Mediterranean and Black Sea stocks, respectively, have rarely used integrated stock assessment models. Thus, biological reference points (BRPs) are typically derived outside the model (see ICES 2022a) and do not, with few exceptions (Masnadi et al. 2021; ICES 2022b) account for DD growth. The use of such reference points for Northeast Atlantic stocks has recently been challenged by Sparholt et al. (2021) who suggest that not accounting for DD growth can negatively bias F_MSY_, positively bias B_MSY_ and B_0_, and consequently lead to foregone surplus yield from the anticipated compensatory biomass growth at low abundance. Furthermore, the authors advocate that only reference points derived from production models, which implicitly account for all sources of DD growth, should be used in fisheries management (Sparholt et al. 2021). Such findings highlight the need for more research on the effects of DD growth on the KPPs used in stock assessment and advice.

The effects of DD on fish stocks are expected to be complex. This is because DD can affect multiple KPPs that can covary (e.g., growth, maturation and natural mortality) and DD responses are likely to vary on a stock‐by‐stock basis. For example, a recent study by Rindorf et al. (2022) shows that DD affects the growth of older age classes in more than 50% of Northeast Atlantic stocks, while they found no signs of DD in early growth (i.e., recruits). In most stock assessment models, DD is assumed to impact recruitment, such that the production rate generally decreases as stock size increases (e.g., Beverton and Holt 1957; Ricker 1954; Cadigan 2013). However, Cury et al. (2014) showed that parental biomass is a predictor of only 5%–15% of the variance in recruitment, demonstrating the weak predictive power of the stock–recruitment relationship in marine fish populations. On the other hand, somatic growth variation can be as important as early life‐history survival in driving biomass fluctuations in some marine fish species (Stawitz and Essington 2019) and has direct implications for fisheries advice and sustainable yields.

Here, we expand on recent analyses by testing for the instantaneous effects of density on individual growth in the same year, hereafter referred as instantaneous DD growth (i.e., average weight of the recruits (W_recr_), average weight of the adults (W_old_) and average weight of the population (W_std_)) in 81 stocks from the Northeast Atlantic ICES region. In doing so, we address the following research questions, all of which are explicitly linked to the derivation of reference points used in fisheries management: (1) How many stocks have historically experienced instantaneous DD growth and do stocks of the same species or taxonomic order display similar trends? (2) Is there a common and generalisable instantaneous DD growth relationship that is common to stocks in the region? And finally, (3) for stocks that exhibit a significant negative instantaneous DD growth effect, can we quantify the strength of the relationship? For operational applications, we consider the strength of the instantaneous DD growth relationship a particularly relevant aspect because a stock might experience significant but very weak DD growth, which in turn will have a negligible effect on BRPs. Unlike Rindorf et al. (2022), we also explicitly test for the presence of temporal autocorrelation in all models, which is assumed to be related to cohort effects, that is cohorts passing through the population over time (Kell et al. 2016).

We first analyse instantaneous DD growth (defined as the process in which population density measured in year *y* will affect weight in year *y*), which is assumed, following Sparholt et al. (2021), to have a direct effect on estimates of reference points for fisheries management. We then compared the results of instantaneous DD growth against intracohort DD growth (see Croll and van Kooten 2022; Croll, van Kooten, and de Roos 2023 and reference therein) in weight‐at‐age, to evaluate whether large cohorts exhibit DD growth. Intracohort DD growth is analysed using two different models, in the first, competition is mainly within a cohort, with individuals competing with members of their own cohort where the growth increment across all ages (or a subset of ages) depends on the initial size of the cohort (W_cohort_). In the second, the abundance of the age class affects the growth rate of the cohort, presumably through competition for food. In this second hypothesis, the growth increment for each age is related to the total number of that age (W_cohort_age_).

Thus, we test a fourth research question: (4) Is DD growth operating as an intracohort process as opposed an instantaneous effect? This process is fundamentally different from DD *sensu* Sparholt et al. (2021) and cannot be directly translated into reference points for fisheries management.

We excluded density‐dependent recruitment from our analysis as Rindorf et al. (2022) have recently shown that density‐dependent recruitment occurs in 68% of the stocks analysed, which increases to 78% when excluding pelagic stocks exhibiting significant trends in spawning stock biomass. We have also excluded direct DD in natural mortality and maturity as time varying estimates of natural mortality and maturity are available only for few stocks in the Northeast Atlantic (ICES 2022a). Furthermore, we have collated new estimates of *B*_0_ and *R*_0_ (i.e., spawning stock biomass and recruitment in the absence of fishing), which represent the theoretical level of stock biomass and recruitment abundance at which DD growth effects should be maximised.

## 2 Methods

The different research questions are addressed using alternative model formulations that link the response variable (i.e., weight) to relative estimates of SSB and R, which represent a proxy for DD growth that is comparable among all stocks. This approach allows regression models to be used to explain time variability in the response variables and their dependence on the relative size of the stock or abundance of recruits.

### 2.1 Database

The data set used in this study comprises stock assessment inputs and outputs for 81 stocks from the entire Northeast Atlantic ICES region. The data set was collated in the form of stock objects of the ‘FLStock’ class as defined in the FLR (Kell et al. 2007) framework (Table S1; ICES 2022a). The 81 stocks are all classified by ICES as Category 1 (i.e., age structured analytical stock assessment) and were last assessed by ICES in 2019 (*n* = 12), 2020 (*n* = 63) or 2021 (*n* = 6). In the following text, stocks are referred to by ICES stock IDs; details on the assessment input and outputs of all stocks are provided in the form of an external open access Shiny Application (https://maxcardinale.shinyapps.io/Indicators/; ICES 2022a). The Shiny App also contains a range of plots that visualise various aspects of each stock's population dynamics and demographic characteristics.

**FIGURE 1:**
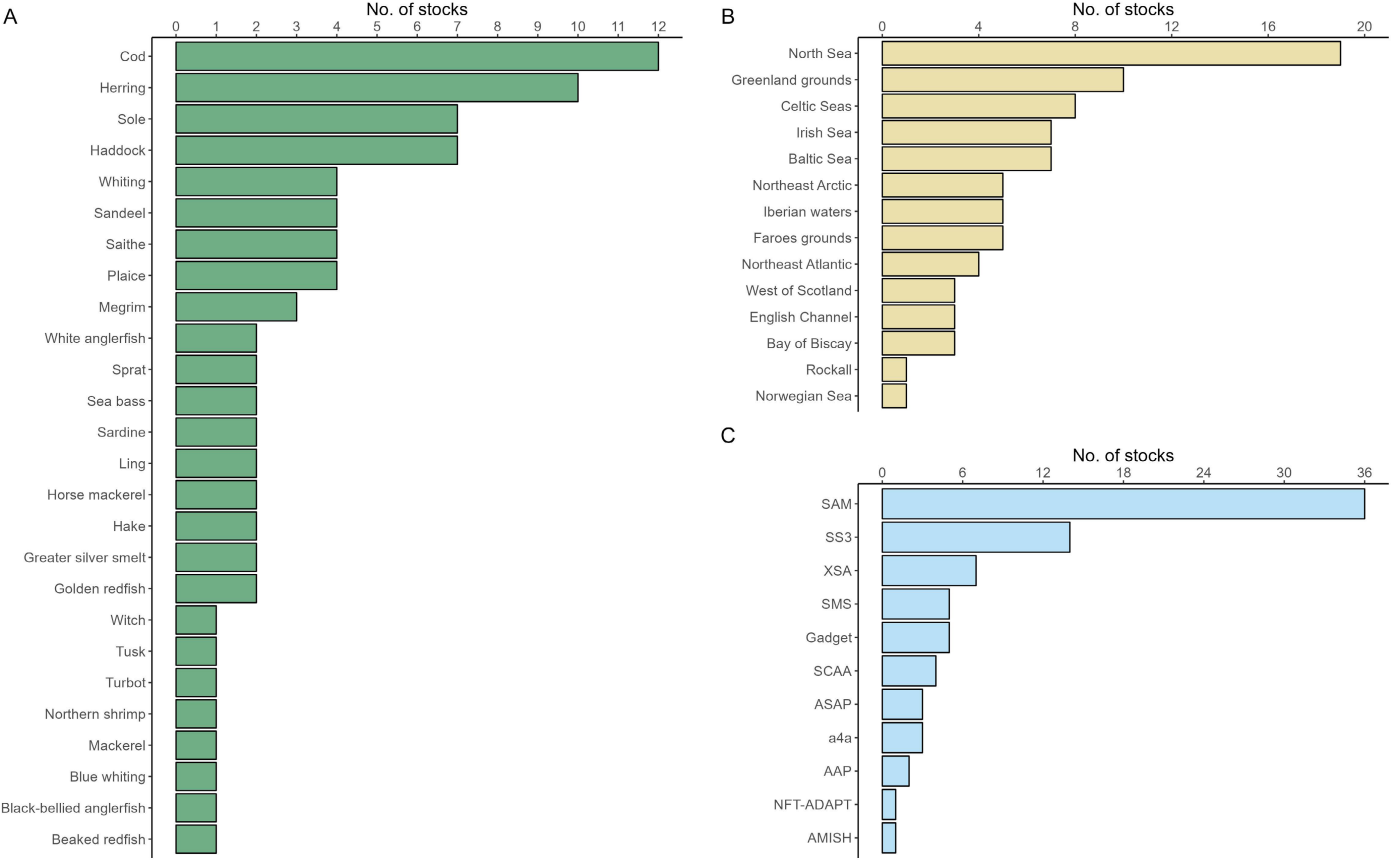
Graphical summary of the dataset used in this study. The dataset contains 26 species (A) from 14 different areas (B), whereby area is a broad characterisation based on ICES areas and ecoregions. All 81 stocks are assessed using age structured analytical assessment frameworks (C), the details of which can be found in Table S1.

A graphical summary of the data set is provided in Figure 1. The data set includes harmonised assessment inputs and outputs from 11 different age‐structured modelling platforms, of which SAM (*n* = 36; Nielsen and Berg 2014) and Stock Synthesis (*n* = 14; Methot and Wetzel 2013) are the most common (see ICES 2022a for more details). The 81 stocks comprise 25 bony fish species (representative of nine taxonomic orders) as well as one crustacean, *Pandalus borealis* (i.e., pra.27.3a4a). Most stocks belong to the following three taxonomic orders Gadiformes (*n* = 33), Pleuronectiformes (*n* = 16) and Clupeiformes (*n* = 14). Note that there is only one Chondrichthyes species (Northeast Atlantic spurdog; *Squalus acanthias*) that is assessed as category 1 by ICES, however, the assessment of this stock is not included in our database.

For the analysis of the weight of the recruits (W_recr_), 23 stocks were excluded as the weight of the first age class was assumed to be constant (i.e., ank.27.78abd, bss.27.4bc7ad‐h, bss.27.8ab, her.27.20–24, her.27,6a7bc, hke.27.3a46‐8abd, hom.27.2a4a5b6a7a‐ce‐k8, lin.27.5a, mac.27.nea, mon.27.78abd, mon.27.8c9a, ple.27.21–23, pra.27.3a4a, reg.27.1–2, sol.27.7 fg and wit.27.3a47d) or had too few observations (i.e., cod.2127.1f14, cod.27.24–32, had.27.5b, reg.27.561214, sol.27.20–24, usk.27.5a14). Of the 81 stocks, 8 stocks were excluded from the weight of the adults (W_old_) and weight of the population (W_std_) instantaneous growth analysis as weight‐at‐age was assumed to be time invariant for the entire time series and all age classes (i.e., ank.27.78abd, bss.27.4bc7ad‐h, bss.27.8ab, hom.27.2a4a5b6a7a‐ce‐k8, ple.27.21–23, pra.27.3a4a and wit.27.3a47d) or had too few observations (cod.27.24–32). Maturity (MAT) and natural mortality (M) were excluded from any of the single regression analyses as less than 31% of the original observations could be retained after pruning for stocks with constant values, few observations, or for periods with assumed constant proportions of MAT and M. Moreover, periods for which W_recr_, W_old_ and W_std_ were assumed to be constant were also excluded from the instantaneous growth analysis.

For the intracohort DD growth analysis (W_cohort_ and W_cohort_age_), 8 of the 81 stocks were excluded as weight‐at‐age was assumed to be time invariant for the entire time series and all age classes (i.e., ank.27.78abd, bss.27.4bc7ad‐h, bss.27.8ab, hom.27.2a4a5b6a7a‐ce‐k8, ple.27.21–23, pra.27.3a4a and wit.27.3a47d) and one stock was excluded because the estimate of R_0_ was not available (i.e., her.27.6a7bc). Moreover, periods for which W_cohort_ and W_cohort_age_ was assumed to be constant were also excluded from the intracohort DD growth analysis.

For each stock, we estimated SSB_0_ by running stochastic simulations to equilibrium at *F* = 0 using the software EqSim (ICES, 2015; https://github.com/wgmg/msy), which is the standard tool used within ICES to estimate reference points. SSB_0_ is the spawning stock biomass under unfished conditions, and it is dependent on assumptions about the stock‐recruitment relationship (SRR), the weight‐at‐age, the maturity‐at‐age and the natural mortality. Unlike other BRPs, such as B_MSY_, calculation of SSB_0_ is independent of assumptions about the selectivity of the fishery and so represents an ideal candidate for our analyses. SSB_0_ is not officially reported by ICES but can be derived from an age structured assessment model if a stock–recruitment relationship is assumed or estimated. In age‐structured models, SSB_0_ is the unfished spawning biomass that is given by the product of virgin recruitment R_0_ and the unfished spawner biomass per recruit (SPR_0_) being it a function of weight‐at‐age, maturity‐at‐age and natural mortality. Like B_MSY_, it is therefore an implicit property of any age‐structured model for which a SRR is estimated or assumed, but currently not reported in ICES. Thus, from the same simulations, the virgin recruitment R_0_ (implicit to the assumed stock recruitment relationship) was also calculated.

For the simulations, year vectors for natural mortality, weights‐at‐age, maturity‐at‐age and selectivity were set as the average of the last 3 years for all stocks. Recruitment was resampled from each stock's predictive distribution, which is based on parametric models that are fitted to the full historical time‐series. Segmented regression is the most used SRR by ICES. For example, during WKMSYREF4 (ICES 2017) mainly segmented regression SRRs were used to derive BRPs to be compatible with precautionary considerations. Thus, we used only segmented regression SRR functions in the simulations with the breakpoint set at the B_lim_ value provided by the ICES 2021 advice (ICES 2022a) to align the estimated SSB_0_ and R_0_ to the stock dynamics as estimated by ICES for each stock. In ICES, B_lim_ is a deterministic biomass limit below which a stock is considered to have reduced reproductive capacity. For the assessment error in the advisory year and the autocorrelation in assessment error in the advisory year, ICES default values (i.e., 0.212 and 0.423, respectively) were used (ICES 2017). Simulations were run for 200 years with the last 50 years being retained to compute equilibrium values. Autocorrelation of recruitment was used in all EqSim simulations.

### 2.2 Relative Estimates of SSB and R, and Weight‐At‐Age

For comparability among stocks, SSB and R were standardised in relation to the biomass and recruitment of the unfished populations (i.e., SSB_0_ and *R*_0_). Thus, we used annual ratios of SSB_y_/SSB_0_ as a relative measure. The same rationale was used for deriving the relative abundance of recruits R (in number of individuals), and thus, the annual ratios of *R*_y_/*R*_0_ were used. This allowed us to consider all stocks in a single model.

For the analysis of the instantaneous DD growth, we used annual weight‐at‐age of recruits (i.e., the youngest age available for each stock) as an indicator of the average weight of the recruits (W_recr_) following the approach by Rindorf et al. (2022). As an indicator of the weight of the adults (W_old_), we first calculated the average age at which 50% of fish achieve first sexual maturity (A_50%_) and then the annual average weight of all age classes above A_50%_. As an indicator of the weight of the population (W_std_), the annual average of all age classes included in the assessment model except the first age class (i.e., recruitment) and the last (i.e., the plus group) was estimated. Both W_old_ and W_std_ were not weighed by the abundance of individuals in the age classes included in the estimation. This is because weighting by the number at age will downweigh considerably the contribution of the old ages and in practice the W_old_ and W_std_ will be mostly influenced by the youngest ages in their respective age intervals. The response of average annual weight‐at‐age in year *y* is not strictly independent of SSB in the same year, because it is used to compute SSB in year *y*. This was already identified by Rindorf et al. (2022). Therefore, here we used the same procedure used in Rindorf et al. (2022) by standardising an equivalent of SSB, using a mean weight‐at‐age across all years when calculating it.

### 2.3 Modelling Instantaneous DD Growth

Generalised additive mixed models (GAMMs) are an extension of generalised linear mixed models (GLMMs) that replace the linear parameter effects with an additive smooth function (Lin and Zhang 1999). The main advantage over GLMMs is that GAMMs can accommodate complex nonlinearities in predictor effects (Hastie and Tibshirani 1986). Furthermore, GAMMs have an advantage over the generalised additive models (GAMs) in that the more complex stochastic structure allows treatment of autocorrelation and repeated measures situations (Wood 2006).

Three alternative GAMM formulations were used to test for the presence of instantaneous DD growth effects on W_recr_, W_old_ and W_std_ across all stocks. Only W_recr_ was related to the continuous predictor variable of relative recruitment strength R/R_0_, whereas the other responses were related to SSB/SSB_0_. If instantaneous DD growth has been historically important for the different stocks, we expect a negative effect of the relative SSB and R on the response variable. Furthermore, if a global effect of instantaneous DD growth exists independently of stock, we assume we would see a negative relationship with the relative SSB or R when removing the interaction between relative SSB or R and stock. To quantify the strength of the instantaneous DD growth on the different stocks, we calculated the slope of the linear trends between W_recr_, W_old_ and W_std_ and relative SSB or R for each stock.

The different models were built following the same structure, that is, from a complex model with a random effect on stock, a fixed effect of species, the effect of the relative SSB or the relative abundance of recruits estimated specifically for each stock, and temporal autocorrelation, to a simple model with only the interaction between the relative SSB or the relative abundance of recruits estimated specifically for each stock and the random effect on stock. The following three model structures were used:

1. (W_recr_, W_old_, W_std_) ~ ((R/R_0_ or SSB/SSB_0_): stock) + species + s(stock, bs = “re”), correlation = corAR1(form = ~year|as factor(stock)
2. (W_recr_, W_old_, W_std_) ~ ((R/R_0_ or SSB/SSB_0_): stock) + s(stock, bs = “re”), correlation = corAR1(form = ~year|as factor(stock)
3. (W_recr_, W_old_, W_std_) ~ ((R/R_0_ or SSB/SSB_0_): stock) + s(stock, bs = “re”)

The effect of the relative SSB or the relative abundance of recruits estimated specifically for each stock was modelled as a linear effect to simplify the interpretation of the instantaneous DD growth relationship. The reason for treating stock as a random effect was because of concerns that multiple observations from the same stock will cause pseudoreplication, which can subsequently result in overestimated precision and significance levels of the model parameters (Thorson and Minto 2015). The error structure of GAMM corrects for the non‐independence of statistical units and permits the ‘random effects’ variance explained at different levels of clustering to be decomposed (Weltz et al. 2013). Similarly, the inclusion of temporal autocorrelation enabled us to account for lack of independence between annual observations within each stock.

The best model was selected using delta AIC (Δi) (Burnham and Anderson 2002, 2004):

$$\Delta_{i}={\mathrm{AIC}}_{i}-{\mathrm{AIC}}_{\min}$$

where AIC_i_ is the AIC estimated for each individual model and AIC_min_ is the lowest AIC estimated among the models tested. Models with Δ_i_ > 10 have essentially no support (Burnham and Anderson 2002) and were discarded. If two or more models had Δ_i_ < 10, the most parsimonious (i.e., with a smaller number of equivalent degrees of freedom) was selected as the best model.

The best model was also used to evaluate the global effect of instantaneous DD growth on each response variable. Specifically, the interaction between the relative SSB or the relative abundance of recruits and the stock was removed from the best model and the global effect of instantaneous DD growth on each response variable was estimated:

4 (W_recr_, W_old_, W_std_) ~ (R/R_0_ or SSB/SSB_0_) +s((R/R_0_ or SSB/SSB_0_), stock, bs="re")

### 2.4 Modelling Intracohort DD Growth

The DD growth cohort effect on weight was calculated from the observed increments in weight along the growth trajectory of each cohort. The differences in the mean individual weight between consecutive ages was calculated for all the ages of a cohort and then standardised by subtracting the mean and dividing by the standard deviation of the weight increment‐at‐age. In this way, these standardised weight increments became comparable along the growth trajectories within each stock, and their average could be calculated over any life period of interest. This was defined as the standardised weight increment by cohort, W_cohort_.

For the W_cohort_ analysis, we took the average of the standardised weight increments by cohort over the ages up to A_50_ and analysed the relationship with the recruitment strength of the cohorts to investigate intracohorts' density‐dependence. This is because during the immature phase, surplus resources are allocated towards somatic growth, while maturity leads to an investment of resources in reproduction (Enberg et al. 2012; Lester, Shuter, and Abrams 2004), and therefore, effects on somatic growth are expected to be stronger for individuals before maturation. We made the recruitment strength of the cohort dimensionless, and so comparable between stocks, using *R*/*R*_0_ where *R* is the number of individuals measured for a given recruitment cohort.

On the other hand, for the W_cohort_age_ analysis, the standardised weight increment by cohort was instead related to the abundance of a given age class estimated the year before. We made the abundance of a given age class dimensionless, and so comparable between stocks, using *N*/*N*_0_ where *N* is the number of individuals measured for given age class and *N*_0_ is the expected number of individuals for the same age class with *F* = 0. This is equivalent to *B*_0_ and *R*_0_ but for an age class so that it is consistent with the instantaneous DD growth analysis. *N*_0_ for each stock and age class were obtained from Griffiths et al. (2023).

We have chosen abundance instead of biomass in the analyses as for many organisms, the relationship between numbers and biomass changes over the life cycle due to changes in individual size, using numbers therefore provides a consistent measure of density over different life stages. This is important for capturing DD acting at specific points in the life cycle, and allows our results to be comparable across teleosts, elasmobranchs and invertebrates. In addition, most classic population dynamics models, like the logistic equation, are formulated in terms of population numbers rather than biomass. Using numbers therefore allows for easier integration with this body of theory and facilitates the estimation of key parameters like carrying capacity and population growth rate (Quinn 2003).

For the analysis of the intracohort DD growth (W_cohort_ and W_cohort_age_), the different models were built following the same structure as for instantaneous growth analysis, that is, from a complex model with a random effect on stock, a fixed effect of species, the effect of the relative cohort strength (*R*/*R*_0_ or *N*/*N*_0_), and temporal autocorrelation, to a simple model with only the interaction between the relative cohort strength (*R*/*R*_0_ or *N*/*N*_0_) estimated specifically for each stock and the random effect on stock. For the temporal autocorrelation, a unique combination of stock and age was used as covariate must have unique values within groups for ‘corAR1’ objects. The following six model structures were used:

1. (Wcohort) ~ ((R/R0): stock) + species +s(stock, bs="re"), correlation = corAR1(form= ~ year | as.factor(stock)
2. (Wcohort) ~ ((R/R0): stock) +s(stock, bs="re"), correlation = corAR1(form= ~ year | as.factor(stock)
3. (Wcohort) ~ ((R/R0): stock) +s(stock, bs="re")
4. (Wcohort_age) ~ (N/N0): stock) + species +s(stock, bs="re"), correlation = corAR1(form= ~ cohort | as.factor(stock:year)
5. (Wcohort_age) ~ (N/N0): stock) +s(stock, bs="re"), correlation = corAR1(form= ~ cohort | as.factor(stock:year)
6. (Wcohort_age) ~ (N/N0): stock) +s(stock, bs="re")

The best model was also used to evaluate the global effect of intracohort DD growth on the response variable. Specifically, the interaction between the relative density of recruits or the biomass of the cohort and the stock was removed from the best model and the global effect of intracohort DD growth was estimated:

7 (W_cohort_, W_cohort_age_) ~ (R/R_0_ or N/N_0_) +s((R/R_0_ or N/N_0_), stock, bs="re")

The analyses were conducted using several libraries on R version 4.2.3.

## 3 Results

### 3.1 Instantaneous DD Growth

All models exhibited significant temporal autocorrelation included as an AR1 process (Table 1). For all models tested, species as a fixed factor was excluded based on delta AIC (Δi) (Table 1), which does not provide evidence that stocks from the same species share a common DD growth process that regulates instantaneous growth. The slope of the stock specific instantaneous DD growth relationship estimated for W_recr_, W_old_ and W_std_ by the best model is shown in Figures S1–S8. Not accounting for temporal autocorrelation has a large effect on the slope of the DD growth relationship (both on the sign and on the strength of the effect), which in extreme cases can change from significantly negative to positive (e.g., cod27.5a and aru.27.5a14 for weight of adults and pok.27.1–2 for W_std_) or from negative to significantly positive (e.g., had.27.46a20 for W_old_) when temporal autocorrelation is accounted for (Figures S1–S6). Visual inspection of the residuals indicated no major departure from model assumptions (Figures S7–S9).

**TABLE 1:** Results of the GAMM on the effect of instantaneous DD growth on the average weight of the recruits (W_recr_), average weight of the adults (W_old_), average weight of the population (W_std_) and intracohort DD growth on weight‐at‐age (W_cohort_ and W_cohort_age_). ‘Species’ is the model with species as fixed effect, ‘No species’ is the model without species as fixed effect and ‘No AR1’ is the model without both species as fixed effect and temporal autocorrelation, ‘Df’ is the degrees of freedom, ‘LogL’ is the log‐likelihood and ‘*n*’ is the number of observations used in the model. The final model selected by Delta AIC is shown in bold.

Response	Df	AIC	LogL	Delta AIC	*r* ^2^	*n*
W_recr_						
Full model	80	5919	−2876	9		
**No species**	**62**	**5909**	**−2891**	**0**	**0.01**	2259
No AR1	61	6400	−3137	490		
W_old_						
Full model	99	6197	−2996	10		
**No species**	**77**	**6187**	**−3015**	**0**	**0.09**	3038
No AR1	76	8361	−4103	2174		
W_std_						
Full model	99	6226	−3010	17		
**No species**	**77**	**6209**	**−3025**	**0**	**0.08**	3032
No AR1	76	8346	−4095	2138		
W_cohort_						
Full model	98	890	−343	26		
**No species**	**76**	**864**	**−354**	**0**	**−0.05**	2822
No AR1	75	4056	−1951	3192		
W_cohort_age_						
Full model	98	52,563	−26,183	24		
**No species**	**76**	**52,539**	**−26,193**	**0**	**−0.02**	22,341
No AR1	75	62,157	−31,003	9618		

**FIGURE 2:**
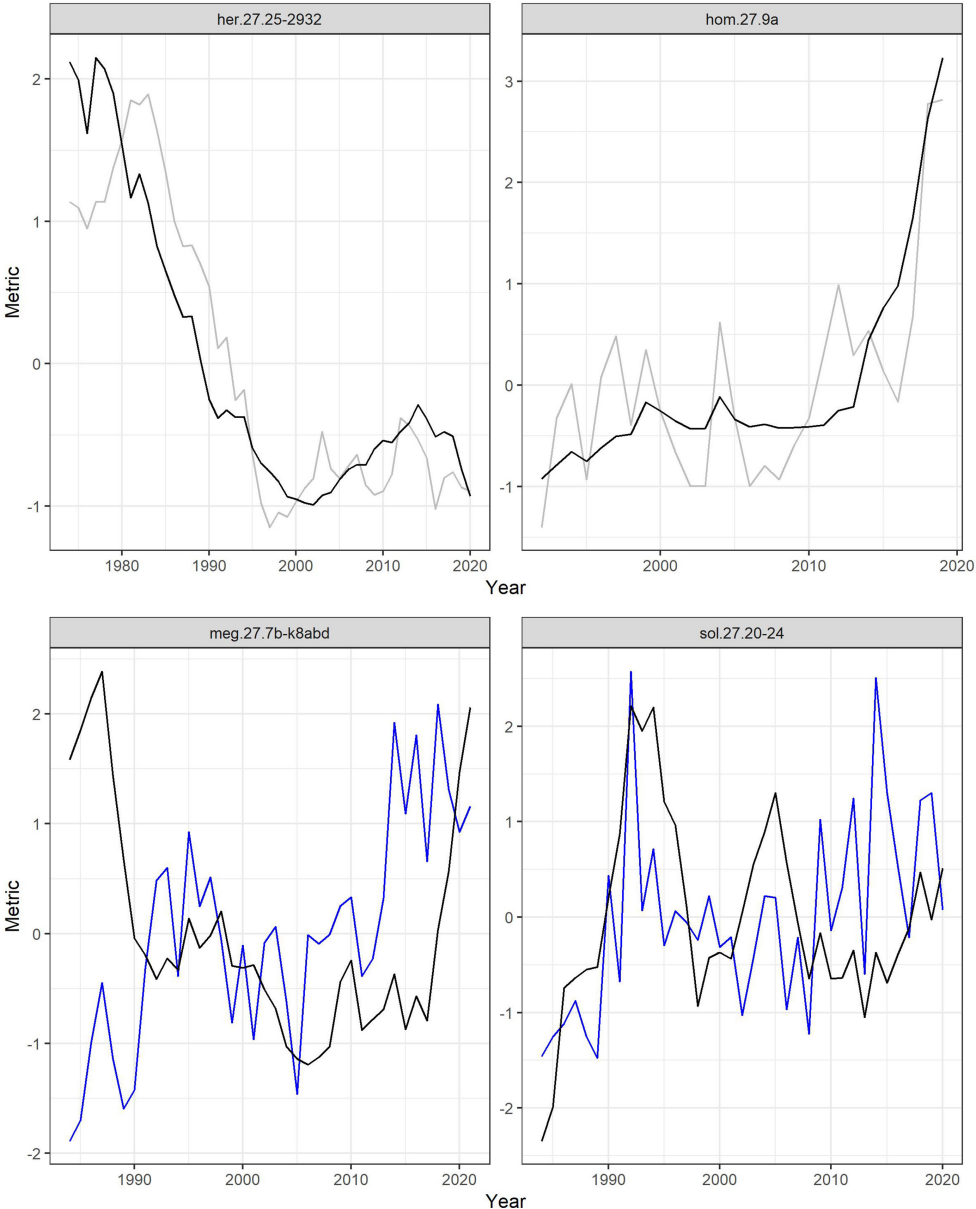
Relationship between the ratio SSB/SSB_0_ (black line; mean standardised) and W_old_ (upper panels, grey line) and W_std_ (lower panels, blue line) for selected stocks which exhibit a significant positive relationship irrespective of whether temporal autocorrelation is accounted for or not.

Figure 2 shows selected stocks that were estimated to exhibit a significant positive relationship between W_old_ and W_std_ and SSB/SSB_0_ irrespective if temporal autocorrelation is accounted for or not.

**FIGURE 3:**
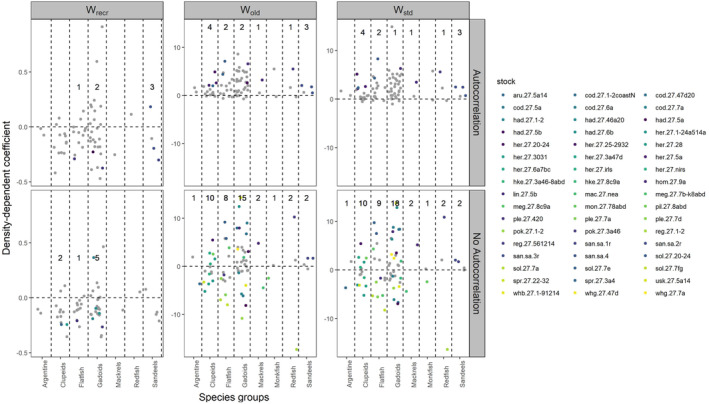
Estimates of the slope of the stock specific instantaneous DD growth for the weight of the recruits (W_recr_), weight of the adults (W_old_) and weight of the population (W_std_) by species groups (Table S1). Coloured dots indicated stocks with significant slopes while grey dots indicate stocks with non‐significant slopes. Top panels are results from the best model, which accounts for temporal autocorrelation, bottom panels are the results from the same model but without accounting for temporal autocorrelation. The number indicates the number of stocks with a significant slope.

When analysing instantaneous DD growth for the different species groups, the frequency of significant relationships generally increases in all taxonomic orders when temporal autocorrelation is not accounted for, except for W_recr_ and for sandeels in W_old_ and W_std_ (Figure 3).

### 3.2 Relationship Strength for the Instantaneous DD Growth

At the stock level, the frequency and strength of significant instantaneous DD growth was apparent only when temporal autocorrelation was not accounted for (Figure 3; Table 2). For all responses, not accounting for temporal autocorrelation increases the number of stocks with a significant relationship except W_recr_ and, for sandeels, W_old_ and W_std_ (Figure 3; Table 3). Also, the strength of the relationship decreases when accounting for temporal autocorrelation (Tables 3 and 4). When temporal autocorrelation is accounted for, most of the stock specific slopes are smaller and several are estimated at values closer to 0, which indicates that instantaneous DD growth is generally absent at the level of biomass historically observed (Figure 3). However, instantaneous DD growth is present, albeit weak, in the weight of the recruits (Figure 3).

**TABLE 2:** Results of the GAMM on the global effect of instantaneous DD growth on the average weight of the recruits (W_recr_), average weight of the adults (W_old_), average weight of the population (W_std_), and intracohort DD growth on weight‐at‐age (W_cohort_ and W_cohort_age_). ‘Estimate’ is the slope of the model with standard error (Std.Error), ‘*p*’ is the *p*‐value. The model is built from the best model in Table 1; see Methods section for details.

Response	Estimate	Std.Error	*p*	*r* ^2^	Model
W_recr_	−0.077	0.026	< 0.001	0.01	Autocorrelation
W_old_	0.865	0.179	< 0.001	0.02	Autocorrelation
W_std_	0.589	0.117	< 0.001	0.04	Autocorrelation
W_cohort_	−0.006	0.015	0.68	0.01	Autocorrelation
W_cohort_age_	−0.056	0.012	< 0.001	0.02	Autocorrelation
W_recr_	−0.094	0.024	< 0.001	0.01	No Autocorrelation
W_old_	−0.148	0.119	0.21	0.02	No Autocorrelation
W_std_	−0.172	0.116	0.14	0.02	No Autocorrelation
W_cohort_	−0.055	0.014	< 0.001	0.01	No Autocorrelation
W_cohort_age_	−0.129	0.016	< 0.001	0.02	No Autocorrelation

**TABLE 3:** Frequency (% and *n*) and strength (meanCoef) of the instantaneous DD growth for the average weight of the recruits (W_recr_), average weight of the adults (W_old_), average weight of the population (W_std_) and intracohort DD on weight‐at‐age (W_cohort_ and W_cohort_age_) by slope (negative or positive) for models with or without temporal autocorrelation for all stocks.

Response	Model	Significance	Slope	*n*	%	meanCoef
W_recr_	Autocorrelation	all	neg	42	72	−0.170
W_old_	Autocorrelation	all	neg	9	12	−0.359
W_std_	Autocorrelation	all	neg	11	15	−0.287
W_cohort_	Autocorrelation	all	neg	10	14	−0.557
W_cohort_age_	Autocorrelation	all	neg	58	81	−0.084
W_recr_	Autocorrelation	all	pos	16	28	0.199
W_old_	Autocorrelation	all	pos	64	88	2.669
W_std_	Autocorrelation	all	pos	62	85	2.573
W_cohort_	Autocorrelation	all	pos	32	42	0.132
W_cohort_age_	Autocorrelation	all	pos	14	19	0.085
W_recr_	No Autocorrelation	all	neg	46	79	−0.149
W_old_	No Autocorrelation	all	neg	35	48	−3.367
W_std_	No Autocorrelation	all	neg	37	51	−3.404
W_cohort_	No Autocorrelation	all	neg	56	78	−0.167
W_cohort_age_	No Autocorrelation	all	neg	60	83	−0.160
W_recr_	No Autocorrelation	all	pos	12	21	0.122
W_old_	No Autocorrelation	all	pos	38	52	4.555
W_std_	No Autocorrelation	all	pos	36	49	4.100
W_cohort_	No Autocorrelation	all	pos	16	22	0.270
W_cohort_age_	No Autocorrelation	all	pos	12	17	0.167

**TABLE 4:** Frequency (% and *n*) and strength (meanCoef) of the instantaneous DD growth for the average weight of the recruits (W_recr_), average weight of the adults (W_old_), average weight of the population (W_std_) and intracohort DD growth on weight‐at‐age (W_cohort_ and W_cohort_age_) by slope for models with or without temporal autocorrelation for stocks with significant relationship only.

Response	Model	Significance	Slope	*n*	%	meanCoef
W_rec_	Autocorrelation	< 0.05	neg	5	9	−0.278
W_old_	Autocorrelation	< 0.05	neg	0	0	NA
W_std_	Autocorrelation	< 0.05	neg	0	0	NA
W_cohort_	Autocorrelation	< 0.05	neg	4	6	−0.288
W_cohort_age_	Autocorrelation	< 0.05	neg	11	15	−0.166
W_rec_	Autocorrelation	< 0.05	pos	1	2	0.183
W_old_	Autocorrelation	< 0.05	pos	12	16	3.807
W_std_	Autocorrelation	< 0.05	pos	13	18	3.492
W_cohort_	Autocorrelation	< 0.05	pos	4	6	0.534
W_cohort_age_	Autocorrelation	< 0.05	pos	1	4	0.260
W_rec_	No Autocorrelation	< 0.05	neg	7	12	−0.199
W_old_	No Autocorrelation	< 0.05	neg	22	30	−4.633
W_std_	No Autocorrelation	< 0.05	neg	20	27	−5.303
W_cohort_	No Autocorrelation	< 0.05	neg	11	15	−0.383
W_cohort_age_	No Autocorrelation	< 0.05	neg	24	33	−0.279
W_rec_	No Autocorrelation	< 0.05	pos	1	2	0.365
W_old_	No Autocorrelation	< 0.05	pos	23	32	5.702
W_std_	No Autocorrelation	< 0.05	pos	21	29	5.577
W_cohort_	No Autocorrelation	< 0.05	pos	5	7	0.745
W_cohort_age_	No Autocorrelation	< 0.05	pos	3	4	0.520

### 3.3 Intracohort DD Growth

The best model for the intracohort DD growth (W_cohort_ and W_cohort_age_) analysis included an AR1 process while species as a fixed factor was excluded based on delta AIC (Δi) (Table 1), which does not provide evidence that stocks from the same species share a common process that regulates intracohort DD growth. The slope of the stock specific intracohort DD growth relationship estimated by the best model is shown in Figures S10–S13. Not accounting for temporal autocorrelation has a large effect on the significance of the DD growth relationship, as this relationship changes for several stocks from significant to nonsignificant (e.g., spr.27.22–32, pol.27.5b and ple.27.7d for W_cohort_ and had.27.6b, her.27.28 and tur.27.4 for W_cohort_age_) when temporal autocorrelation is accounted for (Figures S10–S13). Visual inspection of the residuals indicated no major departure from model assumptions for the model with temporal autocorrelation but significant departure for the model that does not account for temporal autocorrelation, and autocorrelation in the residuals is generally removed when an AR1 term is included in the model (Figures S14–S17).

**FIGURE 4:**
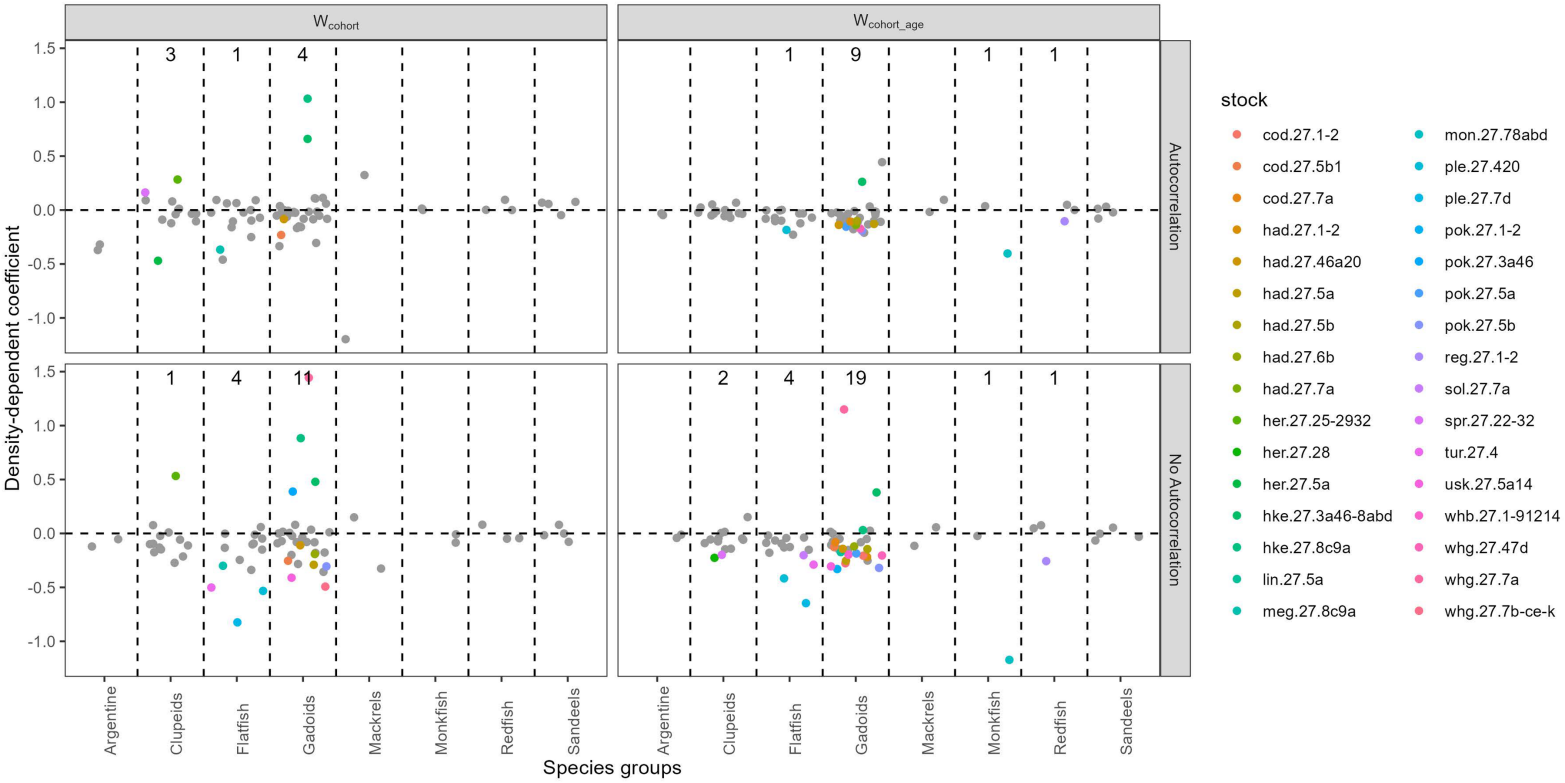
Estimates of the slope of the stock specific intracohort DD growth (W_cohort_ and W_cohort_ages_) by species groups (Table S1). Coloured dots indicated stocks with significant slopes while grey dots indicate stocks with non‐significant slopes. Top panels are results from the best model, which accounts for temporal autocorrelation, bottom panels are the results from the same model but without accounting for temporal autocorrelation. The number indicates the number of stocks with a significant slope.

At the stock level, the frequency and strength of significant intracohort DD growth was more apparent only when temporal autocorrelation was not accounted for (Figure 4; Tables 3 and 4). Not accounting for temporal autocorrelation increases the number of stocks with a significant relationship with the exception of clupeids for W_cohort_ (Figure 4; Table 3). Also, the strength of the relationship decreases when accounting for temporal autocorrelation (Tables 3 and 4). When temporal autocorrelation is accounted for, most of the stock specific slopes are smaller and several are estimated at values closer to 0 and are not significant, which indicates that intracohort DD growth is not a common phenomenon at the level of densities historically observed (Figure 4). W_cohort_age_ shows more significant negative relationships than W_cohort_ although only 15% of the stocks has a significant negative relationship when temporal autocorrelation is accounted for.

When compared to the results of the instantaneous DD growth, more stocks showed significant intracohort DD growth both when temporal autocorrelation was accounted for and when it was not, although, as for the instantaneous DD growth analysis, the number of stocks with significant intracohort DD growth declined greatly when an AR1 term is included in the model (compare Figures 3 and 4).

### 3.4 Global Relationship for DD Growth

The presence of a global instantaneous DD growth relationship for Northeast Atlantic stocks is not generally supported except for W_recr_ (Table 2). Instead, except in the case of W_recr_ a positive relationship between relative SSB and the response variable when temporal autocorrelation was accounted for was shown, while it became non‐significant when the model did not include temporal autocorrelation (Table 2). For intracohort DD growth, W_cohort_ does not show a global DD growth relationship when temporal autocorrelation is accounted for while it is present for W_cohort_age_.

### 3.5 General Tendencies for DD Growth

**FIGURE 5:**
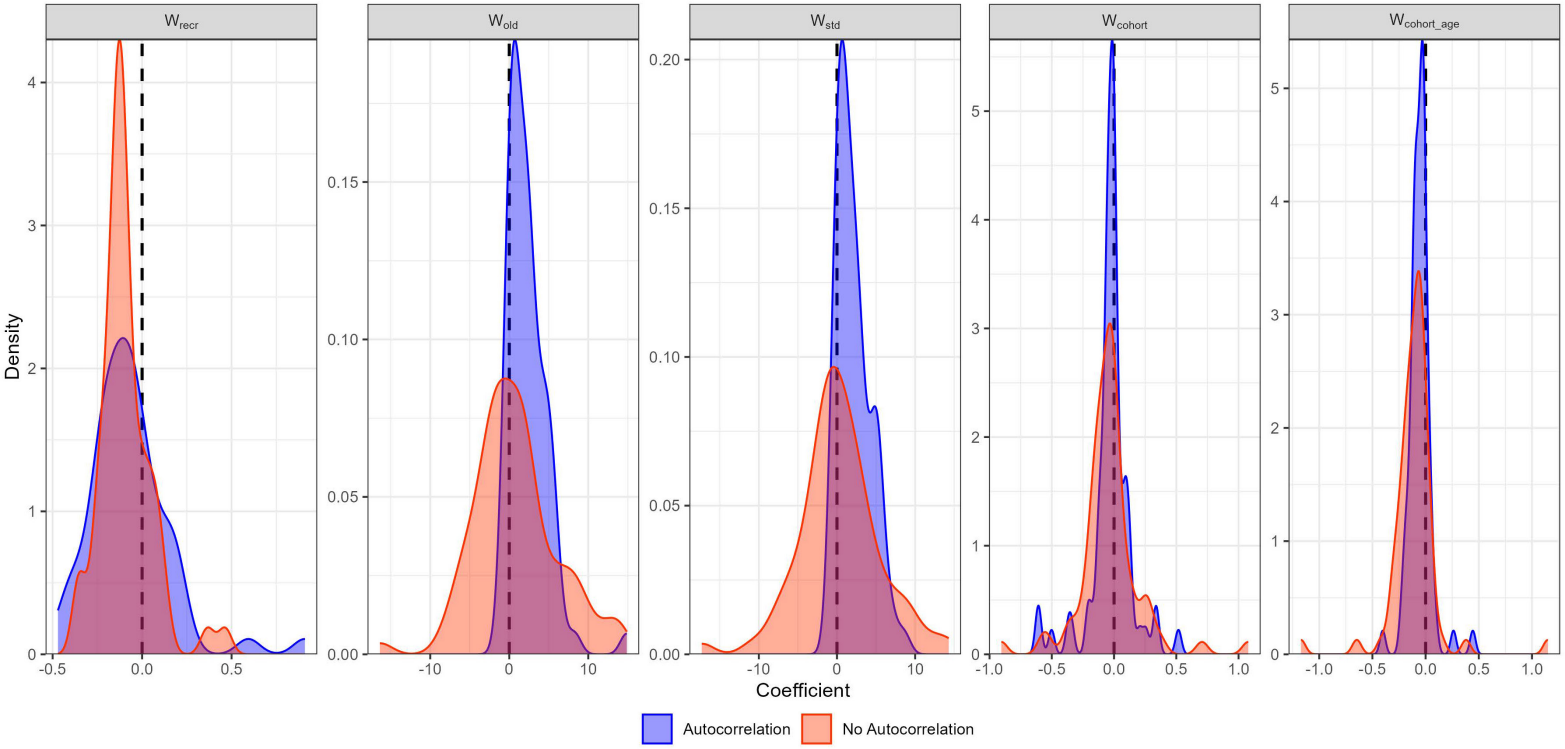
Density plot of the slope of the stock specific instantaneous DD growth for the weight of the recruits (W_recr_), weight of the adults (W_old_), weight of the population (W_std_) and intracohort DD growth (W_cohort_ and W_cohort_age_) with and without temporal autocorrelation.

The results only showed a tendency towards negative slopes and thus a compensatory instantaneous DD growth effect in W_std_ and W_old_ across the analysed fish stocks when temporal autocorrelation was not considered (Figure 5). However, when accounting for temporal autocorrelation, there was an apparent shift towards positive slopes (Figure 5). By contrast, the instantaneous DD growth in W_rec_ showed a distinctive density distribution of estimated slope coefficients, with mode below zero, which also persisted after accounting for temporal autocorrelation. A comparable pattern was evident for intracohort DD growth in W_cohort_ and W_cohort_age_, although the modes shifted slightly more towards zero, while a few stocks even resulted in more negative slope coefficients (Figure 5). As for W_rec_, the tendency for W_cohort_ and W_cohort_age_ was robust to inclusion of temporal autocorrelation in the models (Figure 5).

## 4 Discussion

Our analysis finds no evidence that stocks from the same species share a common instantaneous DD growth process (e.g., stock size in year *y* will affect weight‐at‐age in year *y*), which is implicit to the assumption of increased, compensatory surplus production at low abundance made by Sparholt et al. (2021) and is expected to have a direct effect on estimates of reference points for fisheries management. Furthermore, the analysis also showed that a global instantaneous DD growth cannot be demonstrated in the Northeast Atlantic stocks considered here except for the weight of recruits. On the contrary, two of the three responses tested for instantaneous DD growth showed a global positive effect, suggesting that other processes as opposed to instantaneous DD growth are at work at the level of biomass observed in our time series. At the stock level, when not accounting for temporal autocorrelation, we found instances of apparent instantaneous DD growth in several stocks, particularly the average weight of adults and average weight of the population. However, significant instantaneous DD growth was present only when not accounting for temporal autocorrelation. The results here confirm that, although there has been evidence for instantaneous DD growth in empirical biological data for some fish stocks (e.g., weight‐at‐age; Rindorf et al. 2022), a directional global trend in the Northeast Atlantic cannot be generalised to robustly predict the strength of instantaneous DD growth for any stock (Rindorf et al. 2022; this study).

For intracohort DD growth in weight‐at‐age, the results were similar to those of instantaneous DD growth. The major difference was that a larger proportion of stocks exhibited a classic negative intracohort DD growth compared with instantaneous DD growth; however, the number of stocks with significant intracohort DD growth was generally low and declined further when temporal autocorrelation was considered. These results are in line with the results on Northwest Atlantic herring populations by Beaudry‐Sylvestre, Benoît, and Hutchings (2024), for which the absence of DD growth indicated that the significance of this process may have been overestimated in previous analyses (e.g., Brunel & Dickey‐Collas, 2010). It also indicates that, in general, declines in population density associated with overfishing and/or low recruitment are unlikely to result in compensatory increases in growth rates (Beaudry‐Sylvestre, Benoît, and Hutchings 2024 for Northwest Atlantic herring populations) and weight‐at‐age (this study) across stocks of the Northeast Atlantic. This also points to environmental factors as possible causes of changes in growth (Beaudry‐Sylvestre, Benoît, and Hutchings 2024) or fishing effects that cannot be ruled out (see sections below).

Understanding and recognising the causes of variation in somatic growth has been a major field of research in fisheries ecology and population dynamics (e.g., Beverton and Holt 1957; Lorenzen and Cam 2019). However, although DD growth has been shown to be an important regulating mechanism in the dynamics of some fish populations (e.g., Lorenzen and Enberg 2002; Stenevik et al. 2022), the effects on recruitment typically predominate (Lorenzen 2008; Zimmermann, Ricard, and Heino 2018). Zimmermann, Ricard, and Heino (2018) investigated DD in 70 populations in the Northeast Atlantic and found that DD in recruitment was generally stronger than DD in somatic growth, although in some cases the opposite could also be observed. Zimmermann, Ricard, and Heino (2018) also concluded that detecting DD in somatic growth is highly dependent on the method used. This is consistent with our results, which show that results are sensitive to the inclusion of an autocorrelation term, and when temporal autocorrelation is considered, no global DD growth was found except for the weight of recruits.

Density‐dependent effects might be expected to be stronger as the stock approaches higher biomass levels (i.e., closer to carrying capacity). However, even when using all available assessment data for 81 Northeast Atlantic stocks, DD growth is shown not to be a general phenomenon. Despite this, it is possible that the general absence of DD growth could be an artefact of the SSB and density levels observed during the data period. Current levels of SSB could be too low compared to the range of values at which DD growth regulation may be expected, that is, closer to carrying capacity, B_0_, while DD growth regulation is likely less important when population density is reduced further below carrying capacity (Lorenzen 2008). For example, less than 15% of the stocks considered here have experienced SSB above 50% of B_0_ throughout the available time series. On the other hand, interactions between growth and maturity are complex. Several studies have attributed observed reductions in age at maturity to increases in growth rate (Godø and Moksness 1987) but there is also evidence that early maturation is associated with poor growth where the onset of maturation overrides the size effect (Mayo et al. 1990). Fish populations can respond to a biomass decline with earlier maturity or, conversely, an increasing population with an elevated density can respond with an increasing age and size at maturity (Rose et al. 2001). Besides changes in maturation triggered by fisheries‐induced evolution, the level of plasticity in maturation and reversibility of changes in maturity schedule during the ontogeny remains strongly debated (see Pinsky et al. 2021 and references therein) and heterogeneous spatial fishing pressure might result in changes in growth or maturation simply because it changes the proportion between subcomponents of the same population, which exhibit different phenotypic traits as, for example, growth, condition and maturity.

The results here demonstrated that there is an apparent tendency towards DD growth, which can be detected only when temporal autocorrelation is not considered. Instead, when accounting for temporal autocorrelation and using B_0_ and R_0_ as proxies for carrying capacity, DD growth cannot be generally detected, which implies that generalising DD growth within stock assessment models when estimating reference points could be much less important than recently stated (e.g., Sparholt et al. 2021). This also highlights that the apparent presence of DD growth in current data could be an artefact of temporal autocorrelation. When temporal autocorrelation is accounted for, apparent DD growth relationships become statistically nonsignificant and, in several cases, the general tendency towards DD growth is reversed. Autocorrelation most likely arises due to cohort effects, presumably due to cohorts passing through the population (Kell et al. 2016). The importance of temporal autocorrelation in shaping such relationships was highlighted by Agostini et al. (2008). This is inherently linked to the fundamental issue that when temporal autocorrelation is present, it is not possible to discern between a real mechanistic relation between two variables and the hypothesis that it is correlated to any other process occurring at the same time. Indeed, several other density‐independent processes can cause a decline in growth and conditions in fish. Svedang and Hornborg (2014) showed that selective fishing can induce density‐dependent growth. Kraak et al. (2019), following the basilar work of Lee (1912), showed that size‐selective fishing removes faster‐growing individuals at higher rates than slower‐growing fish, meaning that surviving populations will become dominated by slower growing individuals, which can be confounded with DD growth if the population increases at the same time. Furthermore, genetic effects of fishing can counteract the effect of DD growth; for example, genetic change has been identified as the main explanation for the decline in size and age at maturation of cod cohorts of the late 1980s and early 1990s in Flemish Cap (Rodriguez et al. 2013) and the evolution of growth induced by fishing can occur under sustained and prolonged fishing pressure (Enberg et al. 2012), which is the case for many Northeast Atlantic stocks (e.g., Cardinale et al. 2014). Spatial depletion of local components of an exploited stock may also have occurred more often than currently reported (Cardinale et al. 2011, 2012; Cardinale, Nugroho, and Jonson 2011). If, for example, depleted spatial components were growing larger, weight‐at‐age measured at the stock level, might show artificial correlation with abundance, which in turn can be confused with DD growth. Moreover, changes in growth might arise from other density‐independent processes (Matthias et al. 2018) and factors such as temperature, water clarity, water level, etc. which also play major roles in the variation of somatic growth of fish (e.g., Davidson, Letcher, and Nislow 2010; Claireaux et al. 2022). Decline in condition and growth have also been linked to environmental effects, most notably temperature (Levangie, Blanchfield, and Hutchings 2021; Lindmark et al. 2022) and oxygen (Casini et al. 2016; Roman et al. 2019). Increasing water temperatures are predicted to reduce body size, increase natural mortality and cause earlier age at maturity in many species of marine fish (Levangie, Blanchfield, and Hutchings 2021; Ahti, Kuparinen, and Uusi‐Heikkilä 2020). In all those cases, if any of the mentioned density‐independent processes coincides with an increase in the population, it would be impossible to discern it from a real DD growth effect, as the different processes are temporally correlated.

The study provides valuable insights into the complexity of density‐dependent processes in fish stocks and underscores the importance of evidence‐based fisheries management practices. Although, the study found evidence of instantaneous DD growth in the weight‐at‐age of recruits, indicating a global compensatory effect, other responses did not exhibit a global compensatory effect of DD growth. This suggests that other processes could influence these parameters, such as shifts in productivity and species distribution that could be driven by extrinsic drivers. To manage the associated risks of these uncertainties, adaptive management strategies that are responsive to environmental variability and change are required (e.g., Duplisea et al. 2021; Bentley et al. 2021).

In conclusion, of the responses analysed here, only the average weight‐at‐age of the recruits showed a global instantaneous DD growth effect. However, although the average weight of the recruits shows a general DD growth, the effect is weak and driven by few stocks of gadoids and sandeels. On the other hand, three out of four models showed a positive effect instead of a negative effect, which questions the conclusions of Sparholt et al. (2021) and clearly indicates that other processes are at work that can counteract instantaneous DD growth at the global level. Our results also confirm general conclusions made by Rindorf et al. (2022) and ICES (2022a) that DD growth should be dealt on a stock‐by‐stock basis and cannot be generalised. Surprisingly, the positive effects of large stock size on key productivity parameters such as somatic growth, when temporal autocorrelation is considered, might be more common than previously thought. This indicates that, contrary to common belief, fish populations, and the fisheries that exploit them, might both benefit if stocks were kept at a much higher level of biomass as it favours growth. The asymmetric effects of fishing below or above F_MSY_ are well established in the literature (Beverton 1998; Mace 2001; Hilborn 2010; Hordyk, Huynh, and Carruthers 2019). Restrepo et al. (1998) showed that fishing at just 75% of F_MSY_ would still yield on average more than 95% of MSY based on deterministic age‐structured models that were parameterized with 600 combinations of variations of life history parameters (Mace 1994). In practice, losses in long‐term catches are very small when F is significantly lower than F_MSY_ as also shown by Mace (1994). Thus, fishing down spawning stock biomass with the intent of triggering density dependent mechanisms and thus expecting to gain yields from an exploited population might be associated with far greater risks than keeping biomass at relatively high levels.

## Author Contributions

**Massimiliano Cardinale:** conceptualization (equal), data curation (equal), formal analysis (equal), investigation (equal), methodology (equal), writing – original draft (equal), writing – review and editing (equal). **Valerio Bartolino:** methodology (equal), writing – original draft (equal), writing – review and editing (equal). **Henning Winker:** conceptualization (equal), formal analysis (equal), methodology (equal), writing – original draft (equal), writing – review and editing (equal). **Alessandro Orio:** formal analysis (equal), methodology (equal), writing – review and editing (equal). **Christopher A. Griffiths:** methodology (equal), writing – original draft (equal), writing – review and editing (equal). **Laurie Kell:** writing – original draft (equal), writing – review and editing (equal).

## Conflicts of Interest

The authors declare no conflicts of interest.

## Supporting information


**Figure S1.** Estimated relationship between the average weight of the recruits (W_recr_) and the ratio between R and R_0_ when accounting for temporal autocorrelation. The red asterisk indicated significant relationships at *p* < 0.05.
**Figure S2.** Estimated relationship between the average weight of the recruits (W_recr_) and the ratio between R and R_0_ when not accounting for temporal autocorrelation. The red asterisk indicated significant relationships at *p* < 0.05.
**Figure S3.** Estimated relationship between the average weight of the adults (W_old_) and the ratio between B and B_0_ when accounting for temporal autocorrelation. The red asterisk indicated significant relationships at *p* < 0.05.
**Figure S4.** Estimated relationship between the average weight of the adults (W_old_) and the ratio between B and B_0_ when not accounting for temporal autocorrelation. The red asterisk indicated significant relationships at *p* < 0.05.
**Figure S5.** Estimated relationship between the average weight of the population (W_std_) and the ratio between B and B_0_ when accounting for temporal autocorrelation. The red asterisk indicated significant relationships at *p* < 0.05.
**Figure S6.** Estimated relationship between the average weight of the population (W_std_) and the ratio between B and B_0_ when not accounting for temporal autocorrelation. The red asterisk indicated significant relationships at *p* < 0.05.
**Figure S7.** Residuals (i.e., distribution and standardised autocorrelation function) of the estimated relationship between the average weight of the recruits (W_recr_) and the ratio between R and R_0_ when accounting for temporal autocorrelation.
**Figure S8.** Residuals (i.e., distribution and standardised autocorrelation function) of the estimated relationship between the average weight of the adults (W_old_) and the ratio between B and B_0_ when accounting for temporal autocorrelation.
**Figure S9.** Residuals (i.e., distribution and standardised autocorrelation function) of the estimated relationship between the average weight of the population (W_std_) and the ratio between B and B_0_ when accounting for temporal autocorrelation.
**Figure S10.** Estimated relationship between the weight increment of the cohort (W_cohort_) and the ratio between *R* and *R*_0_ when accounting for temporal autocorrelation. The red asterisk indicated significant relationships at *p* < 0.05.
**Figure S11.** Estimated relationship between the weight increment of the cohort (W_cohort_) and the ratio between R and R_0_ when not accounting for temporal autocorrelation. The red asterisk indicated significant relationships at *p* < 0.05.
**Figure S12.** Estimated relationship between the weight increment of the cohort (W_cohort_age_) and the ratio between N and N_0_ when accounting for temporal autocorrelation. The red asterisk indicated significant relationships at *p* < 0.05.
**Figure S13.** Estimated relationship between the weight increment of the cohort (W_cohort_age_) and the ratio between N and N_0_ when not accounting for temporal autocorrelation. The red asterisk indicated significant relationships at *p* < 0.05.
**Figure S14.** Residuals (i.e., distribution and standardised autocorrelation function) of the estimated relationship between the weight increment of the cohort (W_cohort_) and the ratio between R and R_0_ when accounting for temporal autocorrelation.
**Figure S15.** Residuals (i.e., distribution and standardised autocorrelation function) of the estimated relationship between the weight increment of the cohort (W_cohort_) and the ratio between R and R_0_ when not accounting for temporal autocorrelation.
**Figure S16.** Residuals (i.e., distribution and standardised autocorrelation function) of the estimated relationship between the weight increment of the cohort (W_cohort_age_) and the ratio between N and N_0_ when accounting for temporal autocorrelation.
**Figure S17.** Residuals (i.e., distribution and standardised autocorrelation function) of the estimated relationship between the weight increment of the cohort (W_cohort_age_) and the ratio between N and N_0_ when not accounting for temporal autocorrelation.

Table S1.

## Data Availability

The original data set of stock assessment inputs and outputs is available via ICES as part of the WKREF working group. R code to reproduce the analysis will be made freely available via GitHub (https://github.com/flrpapers) upon acceptance for publication.
