# Supplementary material for: Occurrence and Strength of Instantaneous and Intracohort Density‐Dependence in Northeast Atlantic Fish Stocks
Source: Ecol Evol. 2024 Oct 15;14(10):e70375. doi: 10.1002/ece3.70375 (PMC11480356; doi:10.1002/ece3.70375)
Supplement: Supplementary file 1 — Figure S1. Estimated relationship between the average weight of the recruits (Wrecr) and the ratio between R and R0 when accounting for temporal autocorrelation. The red asterisk indicated significant relationships at p < 0.05. Figure S2. Estimated relationship between the average weight of the recruits (Wrecr) and the ratio between R and R0 when not accounting for temporal autocorrelation. The red asterisk indicated significant relationships at p < 0.05. Figure S3. Estimated relationship between the average weight of the adults (Wold) and the ratio between B and B0 when accounting for temporal autocorrelation. The red asterisk indicated significant relationships at p < 0.05. Figure S4. Estimated relationship between the average weight of the adults (Wold) and the ratio between B and B0 when not accounting for temporal autocorrelation. The red asterisk indicated significant relationships at p < 0.05. Figure S5. Estimated relationship between the average weight of the population (Wstd) and the ratio between B and B0 when accounting for temporal autocorrelation. The red asterisk indicated significant relationships at p < 0.05. Figure S6. Estimated relationship between the average weight of the population (Wstd) and the ratio between B and B0 when not accounting for temporal autocorrelation. The red asterisk indicated significant relationships at p < 0.05. Figure S7. Residuals (i.e., distribution and standardised autocorrelation function) of the estimated relationship between the average weight of the recruits (Wrecr) and the ratio between R and R0 when accounting for temporal autocorrelation. Figure S8. Residuals (i.e., distribution and standardised autocorrelation function) of the estimated relationship between the average weight of the adults (Wold) and the ratio between B and B0 when accounting for temporal autocorrelation. Figure S9. Residuals (i.e., distribution and standardised autocorrelation function) of the estimated relationship between the average weight of [file ECE3-14-e70375-s001.pdf]

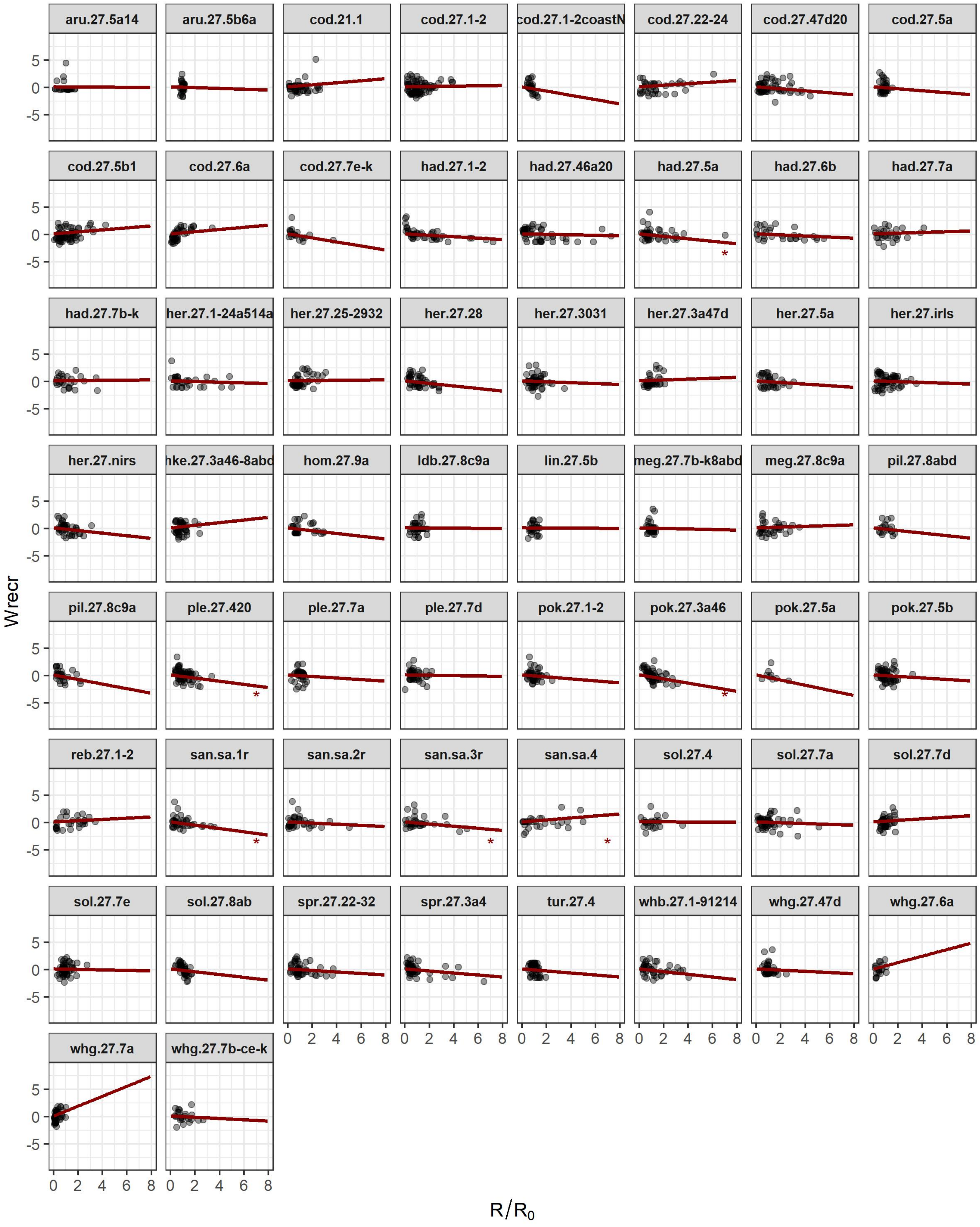
aru.27.5a14
aru.27.5b6a
cod.21.1
cod.27.1-2
cod.27.1-2coastN
cod.27.22-24
cod.27.47d20
cod.27.5a
cod.27.5b1
cod.27.6a
cod.27.7e-k
had.27.1-2
had.27.46a20
had.27.5a
had.27.6b
had.27.7a
had.27.7b-k
her.27.1-24a514a
her.27.25-2932
her.27.28
her.27.3031
her.27.3a47d
her.27.5a
her.27.irls
her.27.nirs
hke.27.3a46-8abd
hom.27.9a
ldb.27.8c9a
lin.27.5b
meg.27.7b-k8abd
meg.27.8c9a
pil.27.8abd
pil.27.8c9a
ple.27.420
ple.27.7a
ple.27.7d
pok.27.1-2
pok.27.3a46
pok.27.5a
pok.27.5b
reb.27.1-2
san.sa.1r
san.sa.2r
san.sa.3r
san.sa.4
sol.27.4
sol.27.7a
sol.27.7d
sol.27.7e
sol.27.8ab
spr.27.22-32
spr.27.3a4
tur.27.4
whb.27.1-91214
whg.27.47d
whg.27.6a
whg.27.7a
whg.27.7b-ce-k
Wrecr
R/R_0

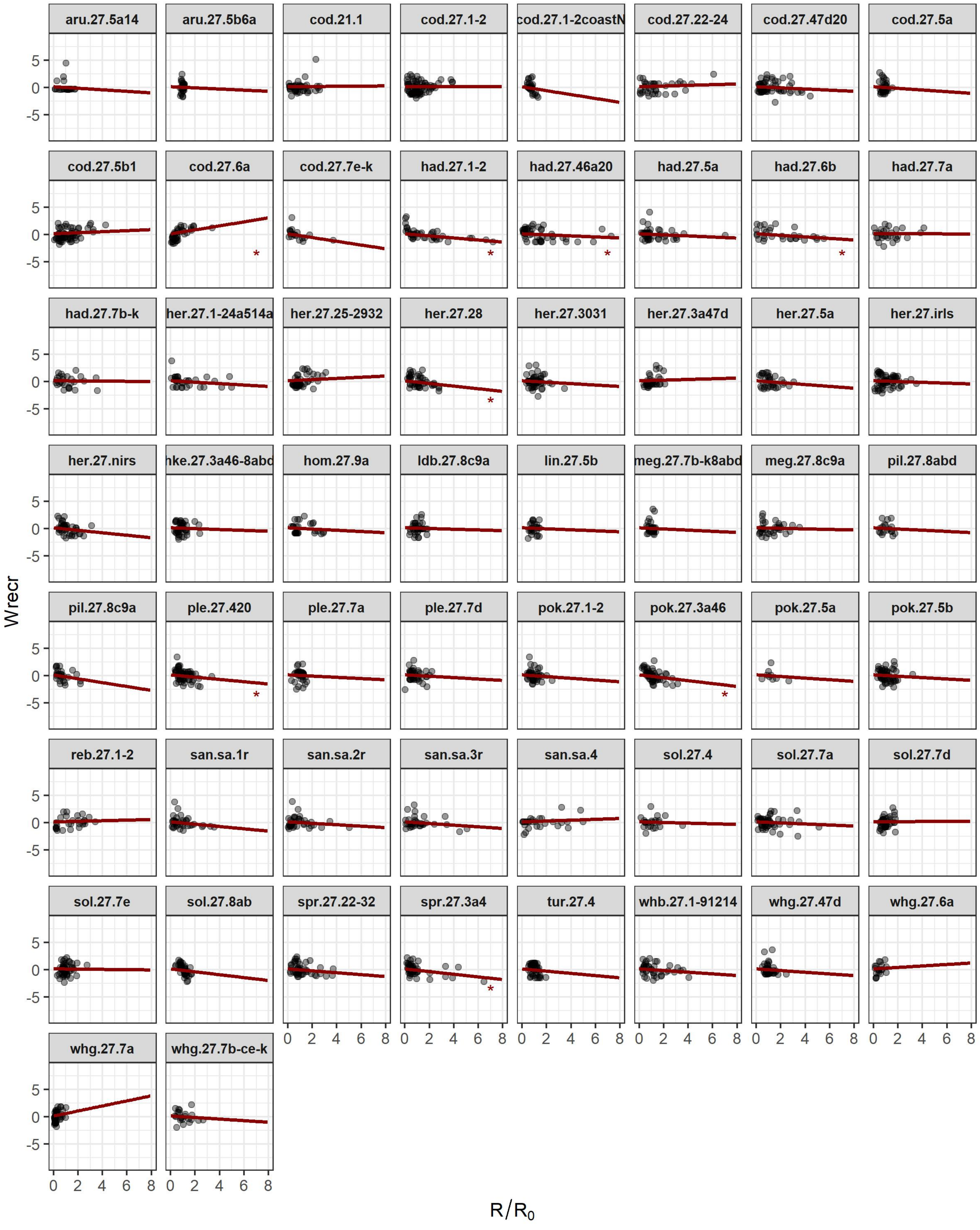
aru.27.5a14
aru.27.5b6a
cod.21.1
cod.27.1-2
cod.27.1-2coastN
cod.27.22-24
cod.27.47d20
cod.27.5a
cod.27.5b1
cod.27.6a
cod.27.7e-k
had.27.1-2
had.27.46a20
had.27.5a
had.27.6b
had.27.7a
had.27.7b-k
her.27.1-24a514a
her.27.25-2932
her.27.28
her.27.3031
her.27.3a47d
her.27.5a
her.27.irls
her.27.nirs
hke.27.3a46-8abd
hom.27.9a
ldb.27.8c9a
lin.27.5b
meg.27.7b-k8abd
meg.27.8c9a
pil.27.8abd
pil.27.8c9a
ple.27.420
ple.27.7a
ple.27.7d
pok.27.1-2
pok.27.3a46
pok.27.5a
pok.27.5b
reb.27.1-2
san.sa.1r
san.sa.2r
san.sa.3r
san.sa.4
sol.27.4
sol.27.7a
sol.27.7d
sol.27.7e
sol.27.8ab
spr.27.22-32
spr.27.3a4
tur.27.4
whb.27.1-91214
whg.27.47d
whg.27.6a
whg.27.7a
whg.27.7b-ce-k
Wrecr
R/R_0

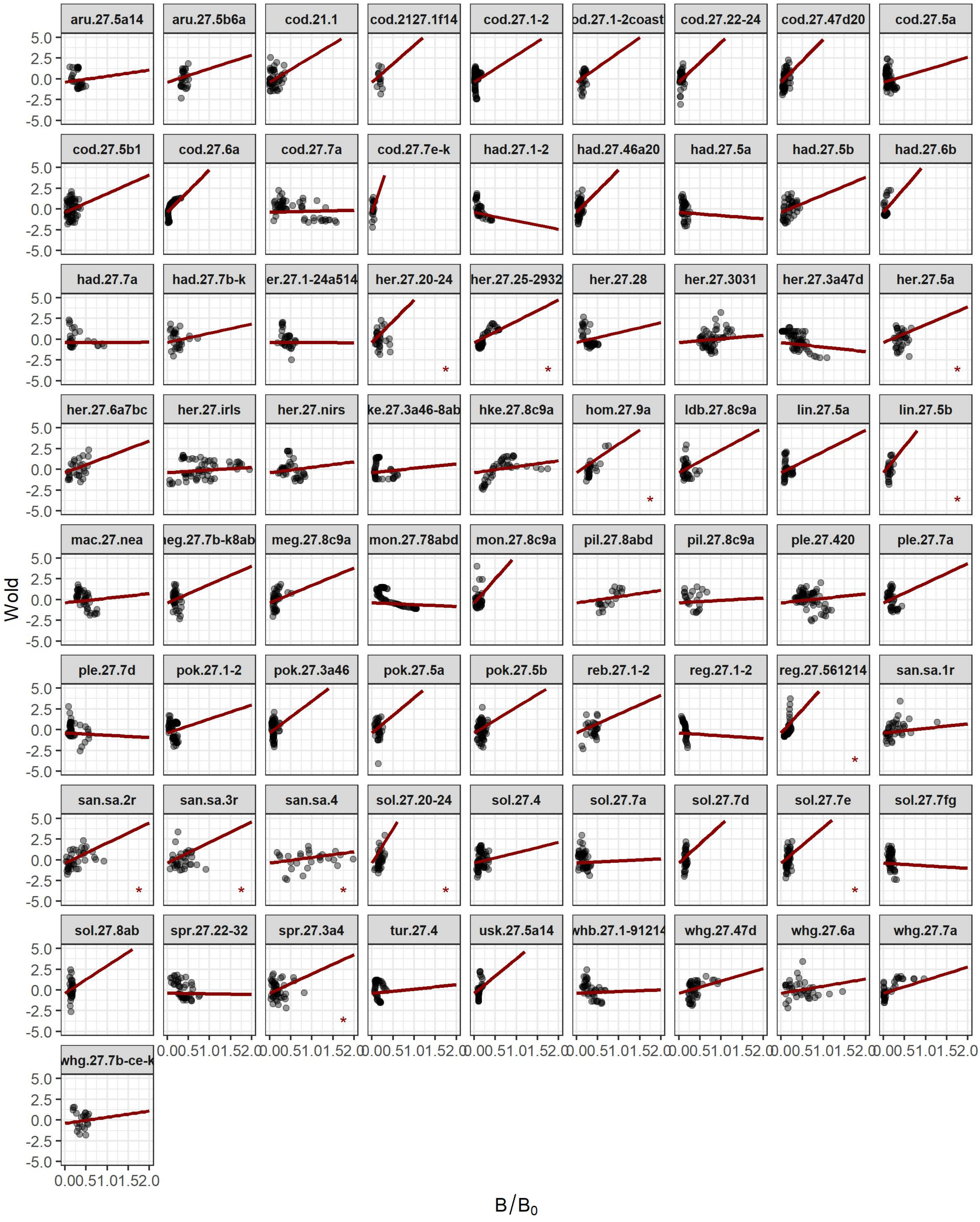
aru.27.5a14
aru.27.5b6a
cod.21.1
cod.2127.1f14
cod.27.1-2
od.27.1-2coast
cod.27.22-24
cod.27.47d20
cod.27.5a
cod.27.5b1
cod.27.6a
cod.27.7a
cod.27.7e-k
had.27.1-2
had.27.46a20
had.27.5a
had.27.5b
had.27.6b
had.27.7a
had.27.7b-k
er.27.1-24a514
her.27.20-24
her.27.25-2932
her.27.28
her.27.3031
her.27.3a47d
her.27.5a
her.27.6a7bc
her.27.irls
her.27.nirs
ke.27.3a46-8ab
hke.27.8c9a
hom.27.9a
ldb.27.8c9a
lin.27.5a
lin.27.5b
mac.27.nea
ieg.27.7b-k8ab
meg.27.8c9a
mon.27.78abd
mon.27.8c9a
pil.27.8abd
pil.27.8c9a
ple.27.420
ple.27.7a
ple.27.7d
pok.27.1-2
pok.27.3a46
pok.27.5a
pok.27.5b
reb.27.1-2
reg.27.1-2
reg.27.561214
san.sa.1r
san.sa.2r
san.sa.3r
san.sa.4
sol.27.20-24
sol.27.4
sol.27.7a
sol.27.7d
sol.27.7e
sol.27.7fg
sol.27.8ab
spr.27.22-32
spr.27.3a4
tur.27.4
usk.27.5a14
whb.27.1-91214
whg.27.47d
whg.27.6a
whg.27.7a
whg.27.7b-ce-k
5.0
2.5
0.0
-2.5
-5.0
0.00.51.01.52.0
Wold
B/B0

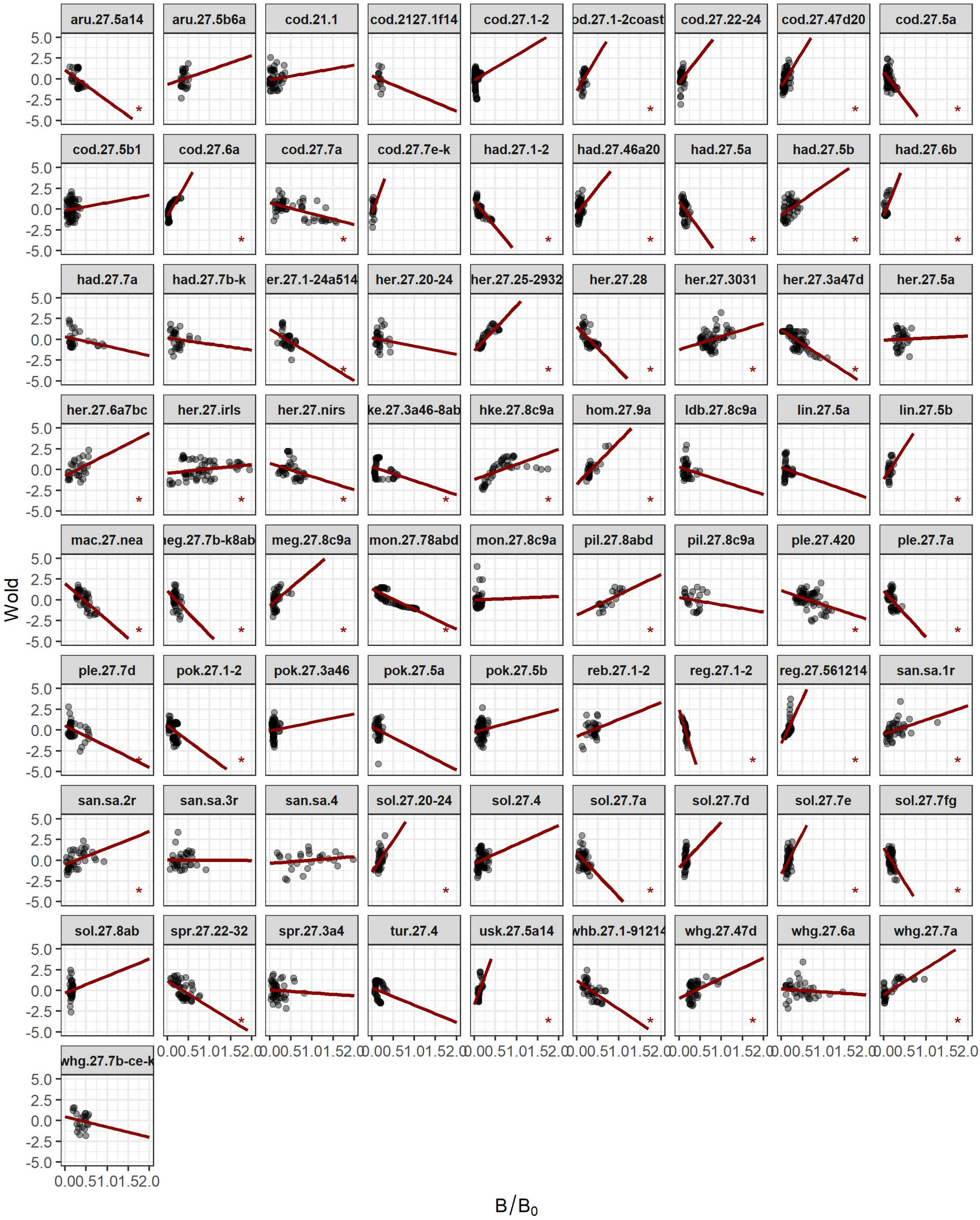
aru.27.5a14
aru.27.5b6a
cod.21.1
cod.2127.1f14
cod.27.1-2
od.27.1-2coast
cod.27.22-24
cod.27.47d20
cod.27.5a
cod.27.5b1
cod.27.6a
cod.27.7a
cod.27.7e-k
had.27.1-2
had.27.46a20
had.27.5a
had.27.5b
had.27.6b
had.27.7a
had.27.7b-k
er.27.1-24a514
her.27.20-24
her.27.25-2932
her.27.28
her.27.3031
her.27.3a47d
her.27.5a
her.27.6a7bc
her.27.irls
her.27.nirs
ke.27.3a46-8ab
hke.27.8c9a
hom.27.9a
ldb.27.8c9a
lin.27.5a
lin.27.5b
mac.27.nea
ieg.27.7b-k8ab
meg.27.8c9a
mon.27.78abd
mon.27.8c9a
pil.27.8abd
pil.27.8c9a
ple.27.420
ple.27.7a
ple.27.7d
pok.27.1-2
pok.27.3a46
pok.27.5a
pok.27.5b
reb.27.1-2
reg.27.1-2
reg.27.561214
san.sa.1r
san.sa.2r
san.sa.3r
san.sa.4
sol.27.20-24
sol.27.4
sol.27.7a
sol.27.7d
sol.27.7e
sol.27.7fg
sol.27.8ab
spr.27.22-32
spr.27.3a4
tur.27.4
usk.27.5a14
whb.27.1-91214
whg.27.47d
whg.27.6a
whg.27.7a
whg.27.7b-ce-k
Wold
B/B0
5.0
2.5
0.0
-2.5
-5.0
0.00.51.01.52.0

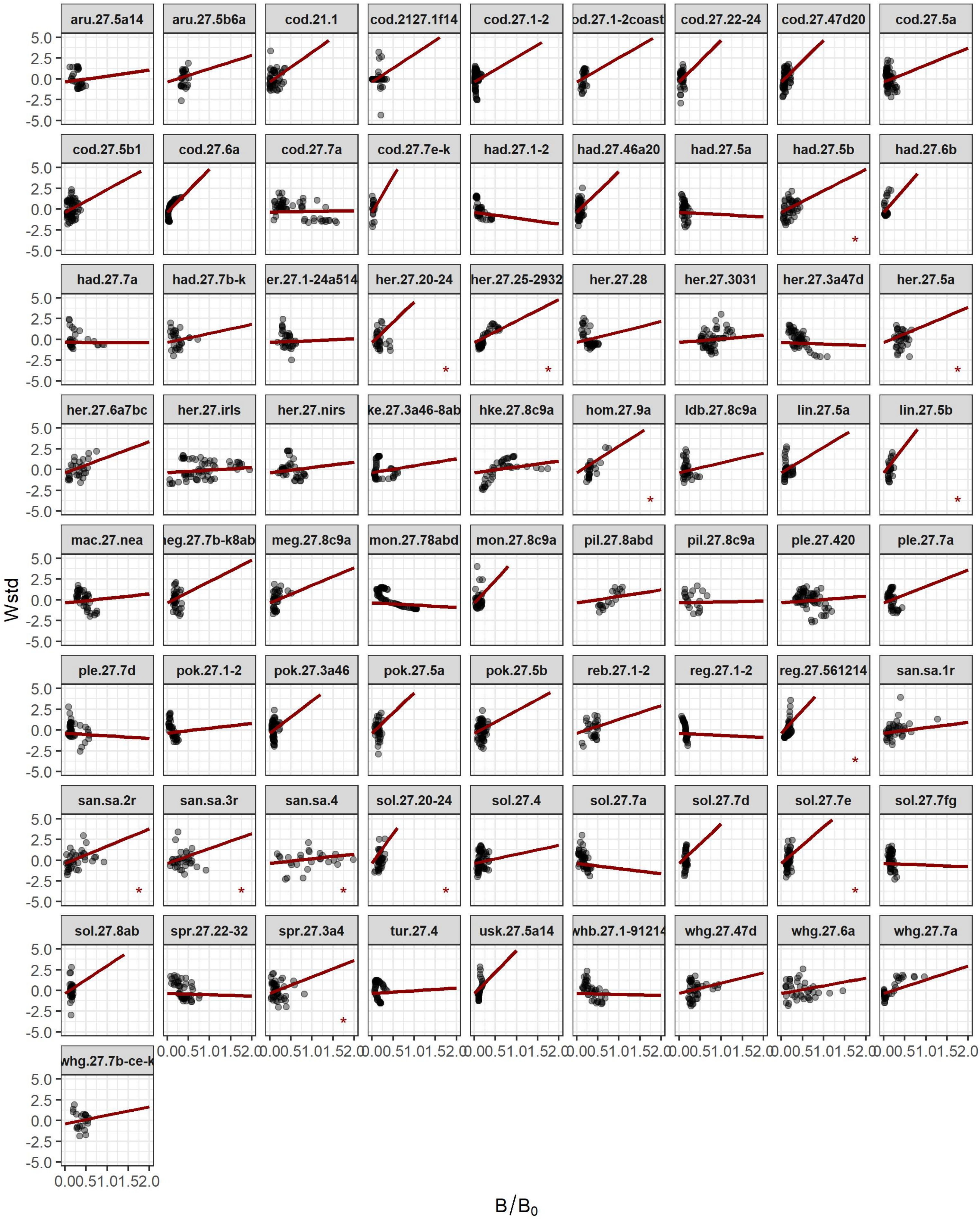

aru.27.5a14
aru.27.5b6a
cod.21.1
cod.2127.1f14
cod.27.1-2
od.27.1-2coast
cod.27.22-24
cod.27.47d20
cod.27.5a
cod.27.5b1
cod.27.6a
cod.27.7a
cod.27.7e-k
had.27.1-2
had.27.46a20
had.27.5a
had.27.5b
had.27.6b
had.27.7a
had.27.7b-k
er.27.1-24a514
her.27.20-24
her.27.25-2932
her.27.28
her.27.3031
her.27.3a47d
her.27.5a
her.27.6a7bc
her.27.irls
her.27.nirs
ke.27.3a46-8ab
hke.27.8c9a
hom.27.9a
ldb.27.8c9a
lin.27.5a
lin.27.5b
mac.27.nea
ieg.27.7b-k8ab
meg.27.8c9a
mon.27.78abd
mon.27.8c9a
pil.27.8abd
pil.27.8c9a
ple.27.420
ple.27.7a
ple.27.7d
pok.27.1-2
pok.27.3a46
pok.27.5a
pok.27.5b
reb.27.1-2
reg.27.1-2
reg.27.561214
san.sa.1r
san.sa.2r
san.sa.3r
san.sa.4
sol.27.20-24
sol.27.4
sol.27.7a
sol.27.7d
sol.27.7e
sol.27.7fg
sol.27.8ab
spr.27.22-32
spr.27.3a4
tur.27.4
usk.27.5a14
whb.27.1-91214
whg.27.47d
whg.27.6a
whg.27.7a
whg.27.7b-ce-k
Wstd
B/B0

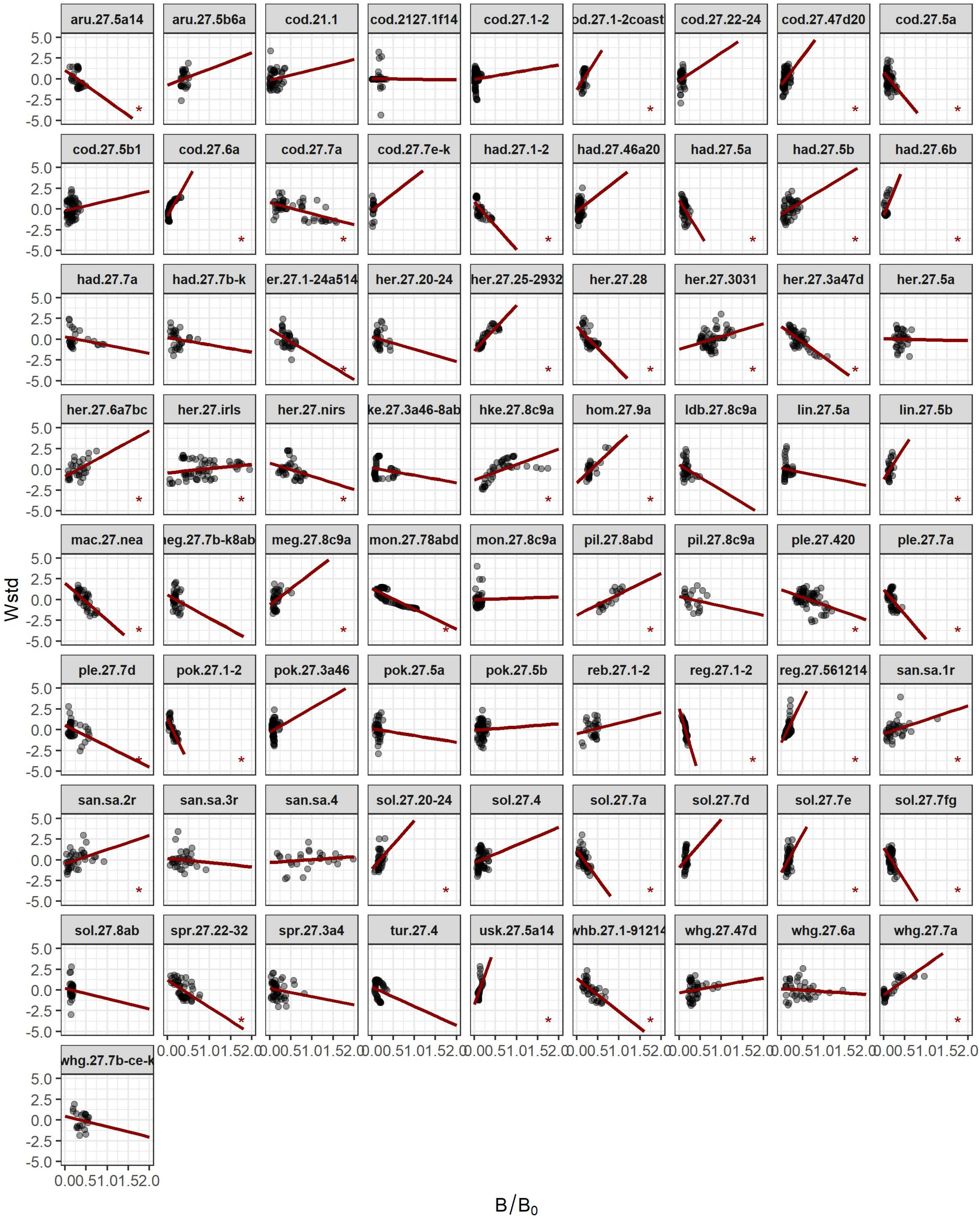

aru.27.5a14
aru.27.5b6a
cod.21.1
cod.2127.1f14
cod.27.1-2
od.27.1-2coast
cod.27.22-24
cod.27.47d20
cod.27.5a
cod.27.5b1
cod.27.6a
cod.27.7a
cod.27.7e-k
had.27.1-2
had.27.46a20
had.27.5a
had.27.5b
had.27.6b
had.27.7a
had.27.7b-k
er.27.1-24a514
her.27.20-24
her.27.25-2932
her.27.28
her.27.3031
her.27.3a47d
her.27.5a
her.27.6a7bc
her.27.irls
her.27.nirs
ke.27.3a46-8ab
hke.27.8c9a
hom.27.9a
ldb.27.8c9a
lin.27.5a
lin.27.5b
mac.27.nea
neg.27.7b-k8ab
meg.27.8c9a
mon.27.78abd
mon.27.8c9a
pil.27.8abd
pil.27.8c9a
ple.27.420
ple.27.7a
ple.27.7d
pok.27.1-2
pok.27.3a46
pok.27.5a
pok.27.5b
reb.27.1-2
reg.27.1-2
reg.27.561214
san.sa.1r
san.sa.2r
san.sa.3r
san.sa.4
sol.27.20-24
sol.27.4
sol.27.7a
sol.27.7d
sol.27.7e
sol.27.7fg
sol.27.8ab
spr.27.22-32
spr.27.3a4
tur.27.4
usk.27.5a14
whb.27.1-91214
whg.27.47d
whg.27.6a
whg.27.7a
whg.27.7b-ce-k
Wstd
B/B0

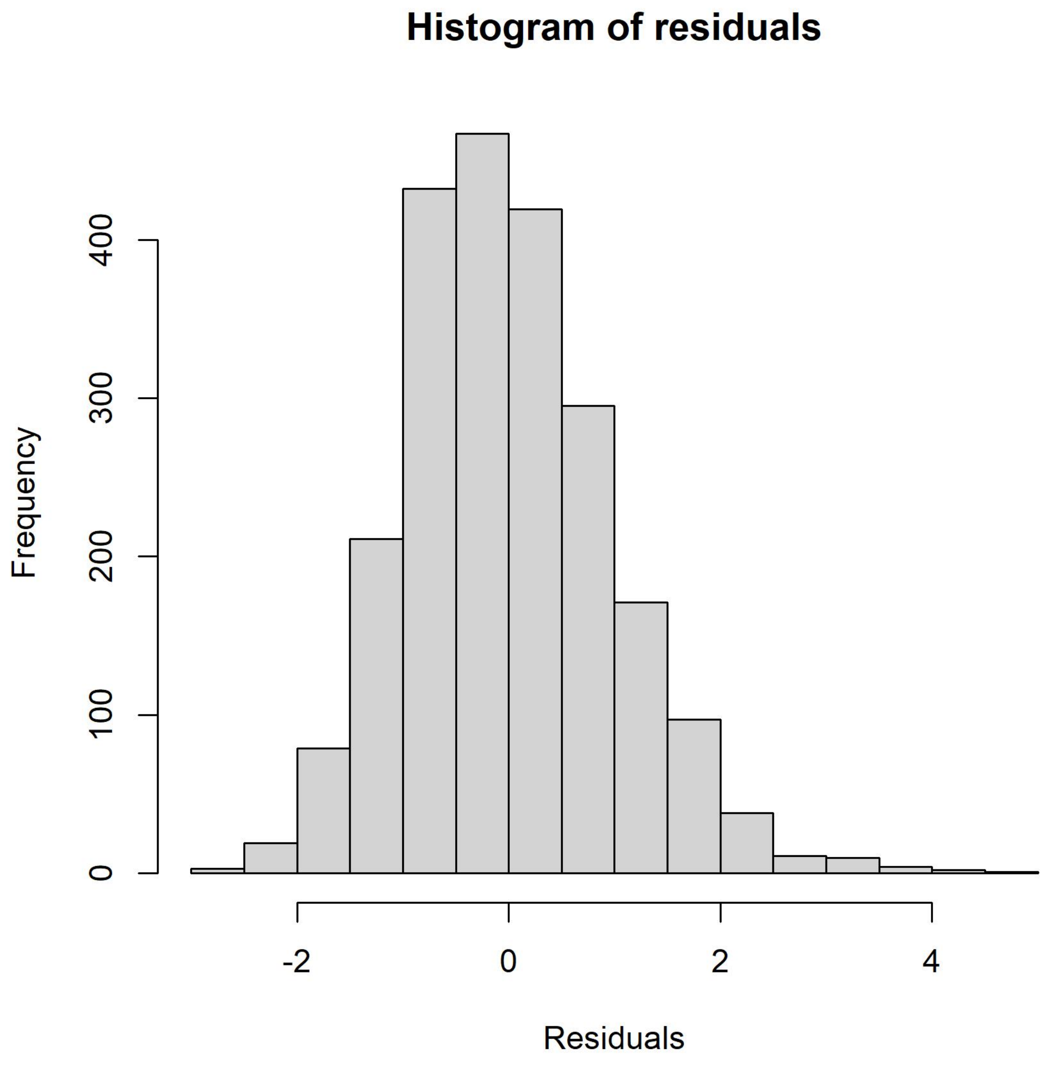
Histogram of residuals
Frequency
Residuals
0
100
200
300
400
-2
0
2
4

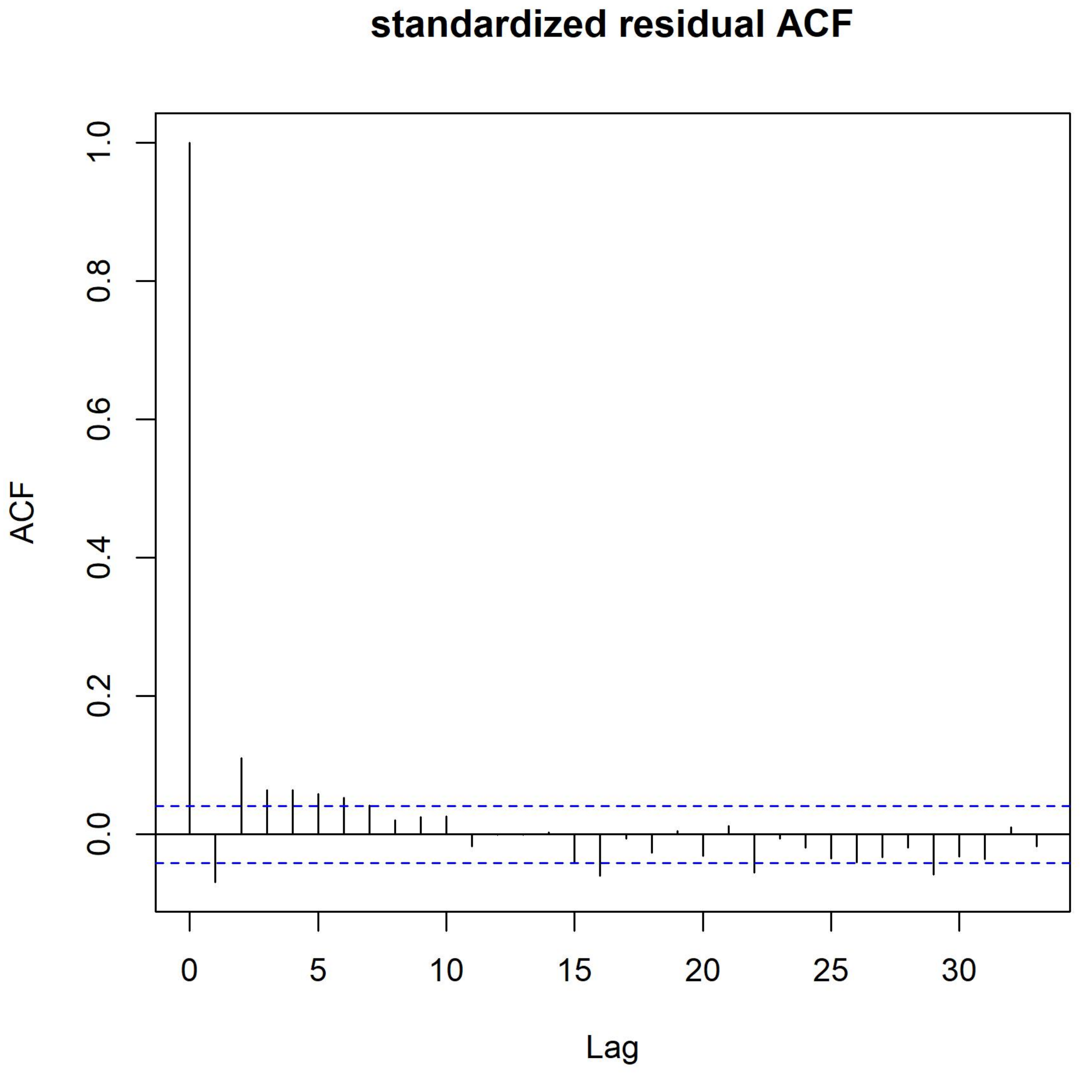
standardized residual ACF
ACF
Lag
0.0
0.2
0.4
0.6
0.8
1.0
0
5
10
15
20
25
30

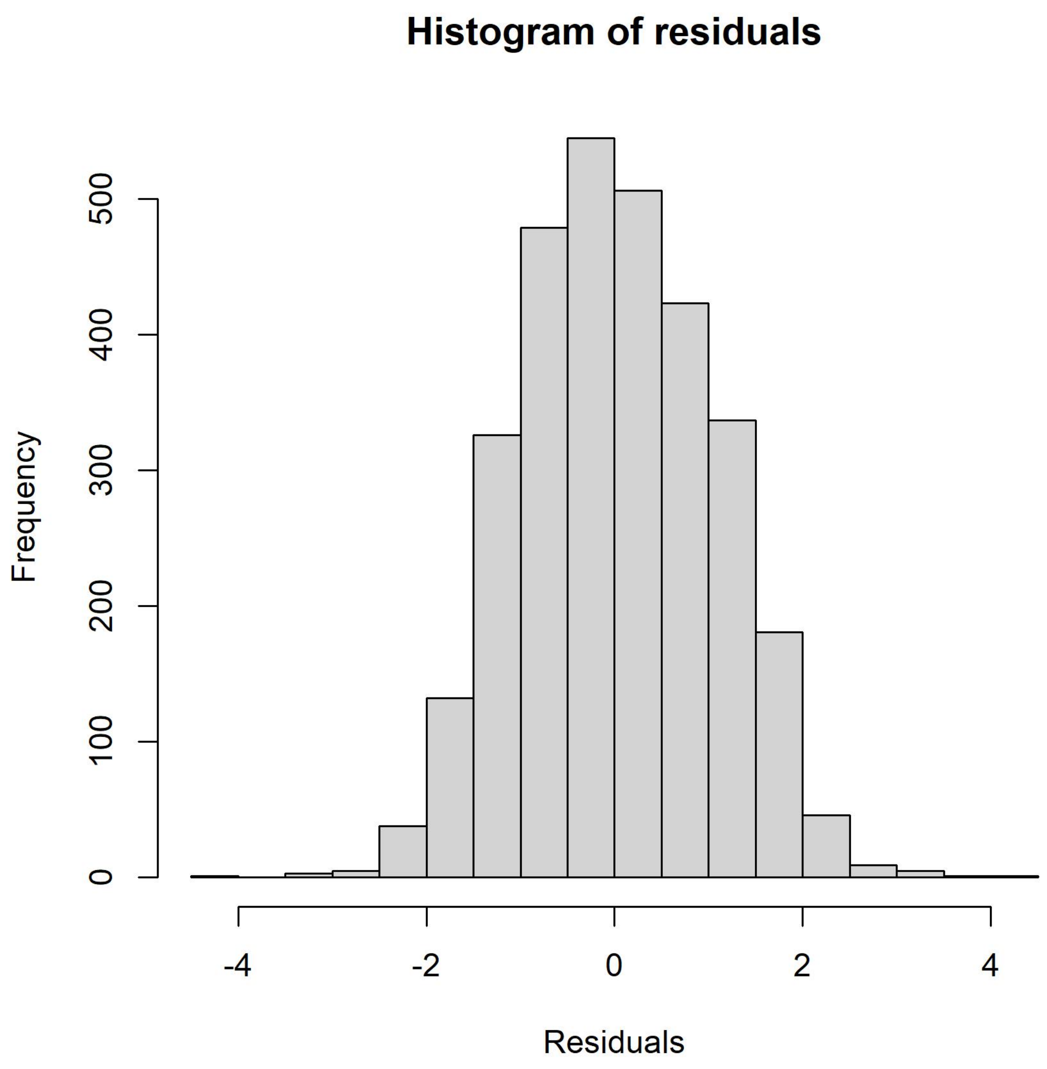
Histogram of residuals
Frequency
0
100
200
300
400
500
-4
-2
0
2
4
Residuals

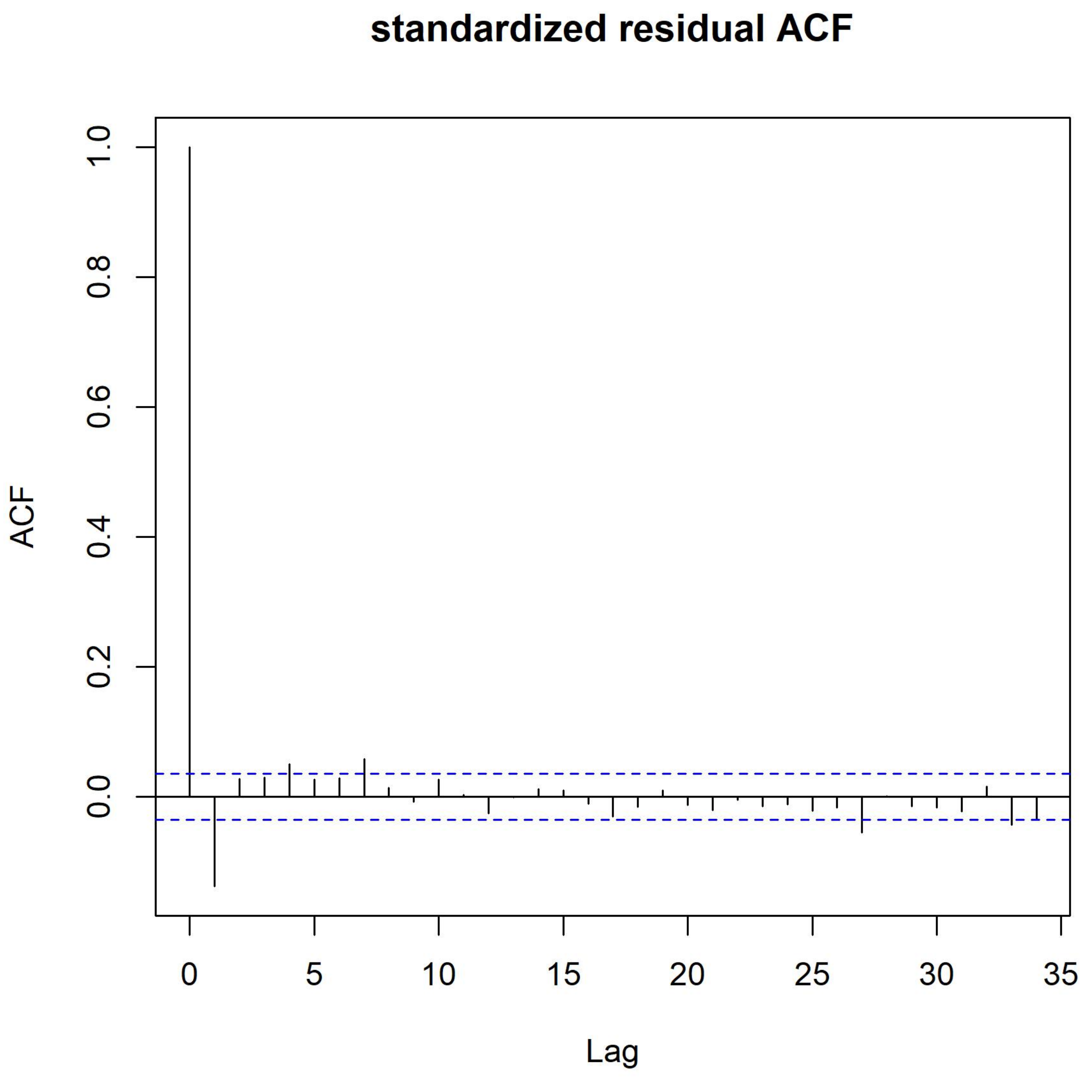
standardized residual ACF
ACF
0.0
0.2
0.4
0.6
0.8
1.0
0
5
10
15
20
25
30
35
Lag

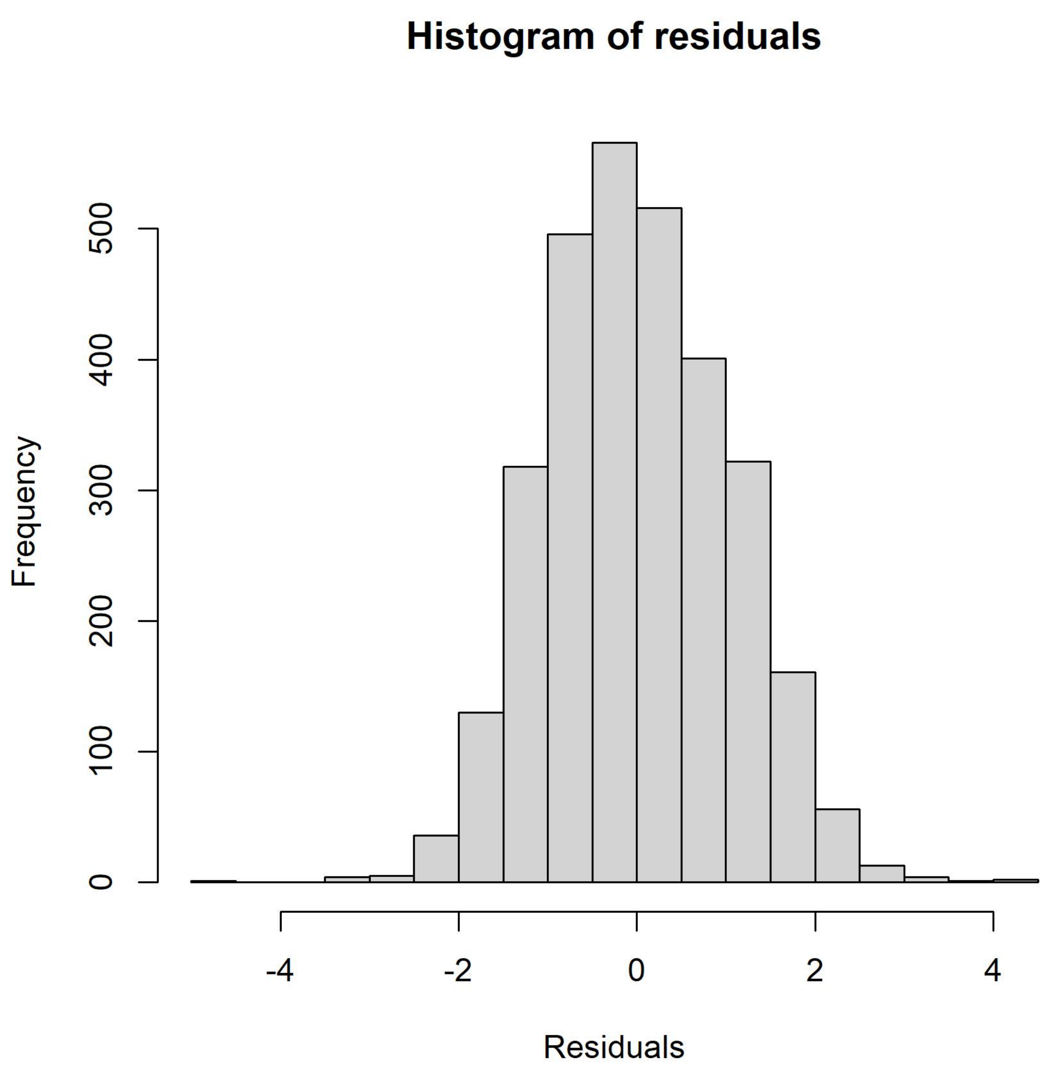
Histogram of residuals
Frequency
Residuals
0
100
200
300
400
500
-4
-2
0
2
4

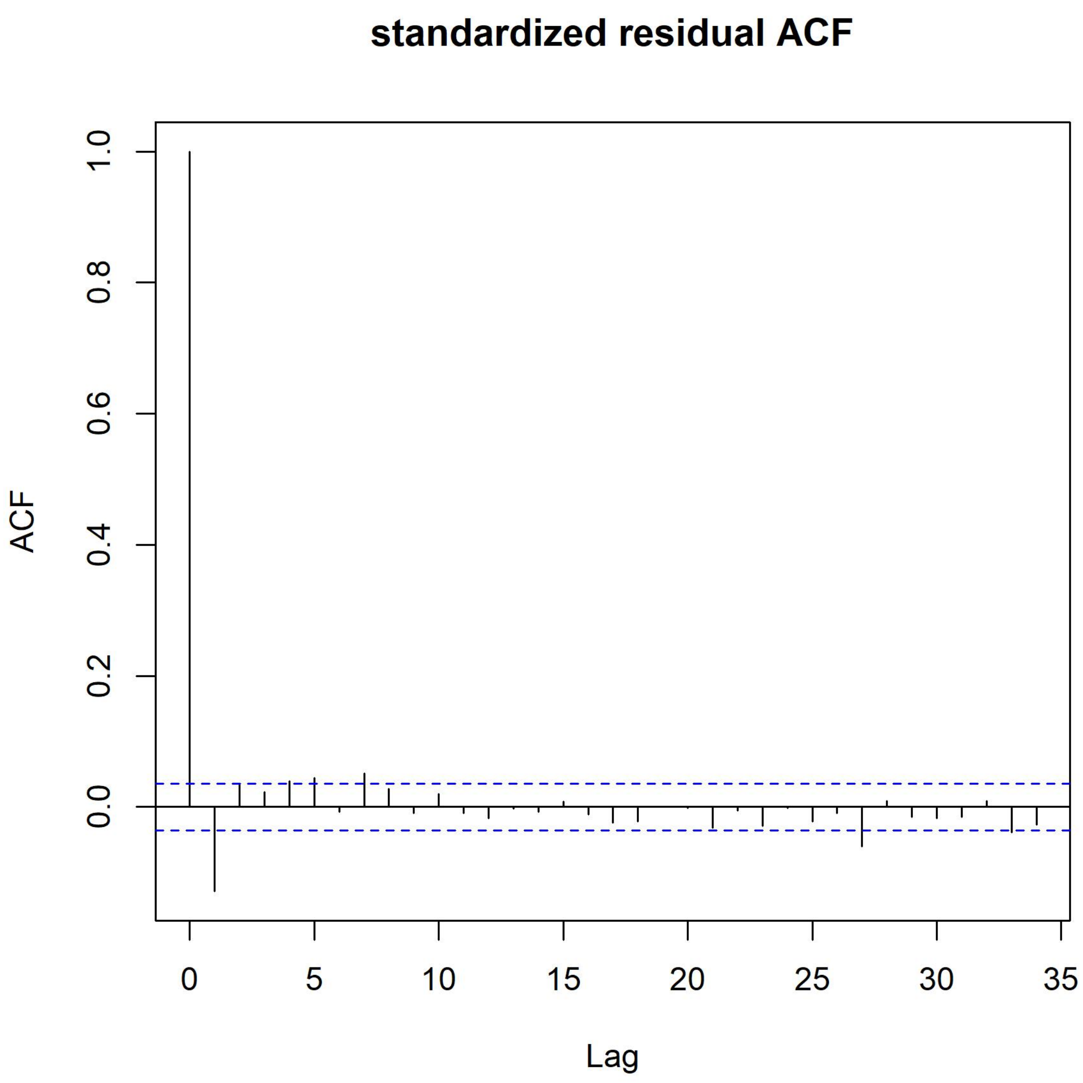
standardized residual ACF
ACF
Lag
0.0
0.2
0.4
0.6
0.8
1.0
0
5
10
15
20
25
30
35

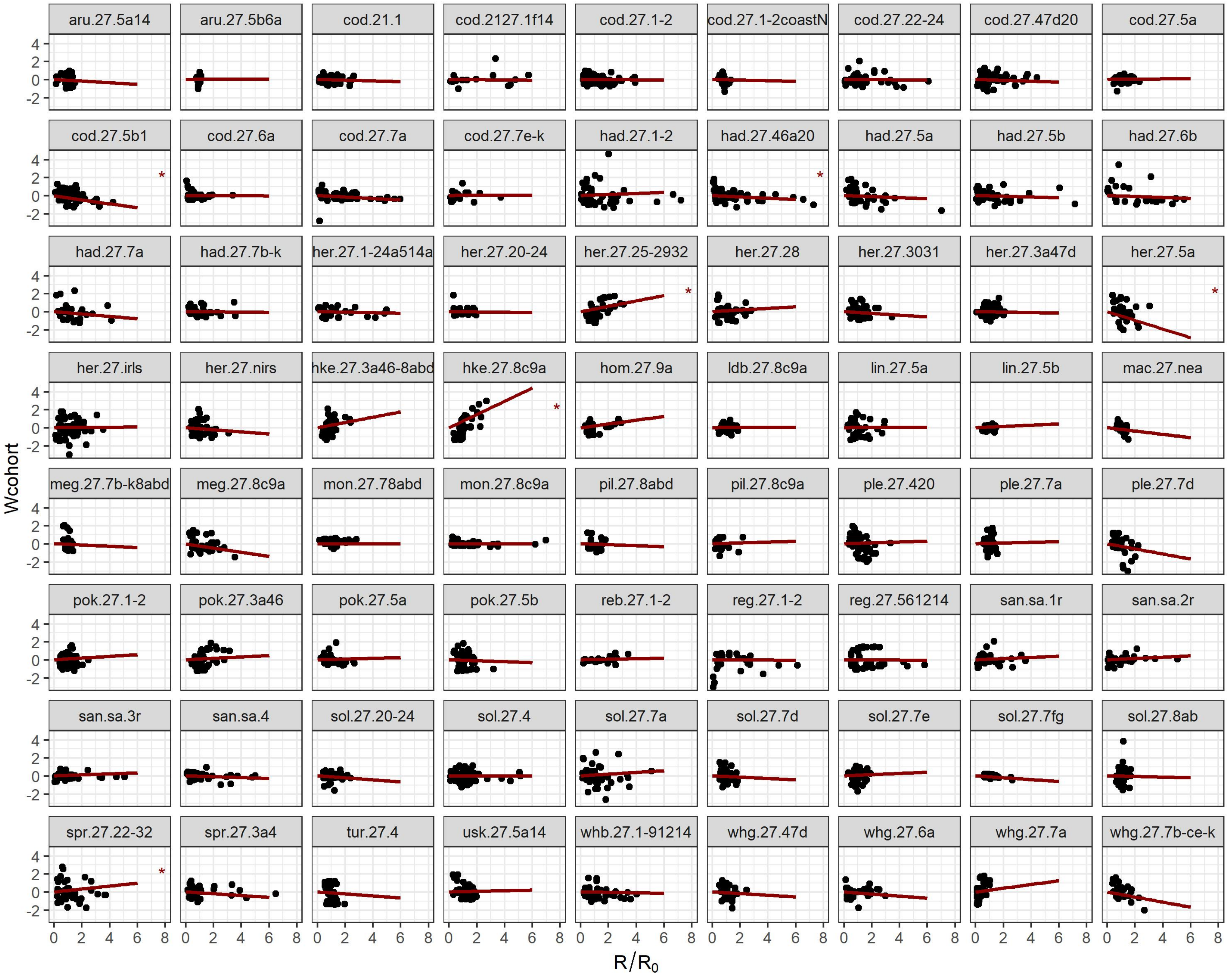
aru.27.5a14
aru.27.5b6a
cod.21.1
cod.2127.1f14
cod.27.1-2
cod.27.1-2coastN
cod.27.22-24
cod.27.47d20
cod.27.5a
cod.27.5b1
cod.27.6a
cod.27.7a
cod.27.7e-k
had.27.1-2
had.27.46a20
had.27.5a
had.27.5b
had.27.6b
had.27.7a
had.27.7b-k
her.27.1-24a514a
her.27.20-24
her.27.25-2932
her.27.28
her.27.3031
her.27.3a47d
her.27.5a
her.27.irls
her.27.nirs
hke.27.3a46-8abd
hke.27.8c9a
hom.27.9a
ldb.27.8c9a
lin.27.5a
lin.27.5b
mac.27.nea
meg.27.7b-k8abd
meg.27.8c9a
mon.27.78abd
mon.27.8c9a
pil.27.8abd
pil.27.8c9a
ple.27.420
ple.27.7a
ple.27.7d
pok.27.1-2
pok.27.3a46
pok.27.5a
pok.27.5b
reb.27.1-2
reg.27.1-2
reg.27.561214
san.sa.1r
san.sa.2r
san.sa.3r
san.sa.4
sol.27.20-24
sol.27.4
sol.27.7a
sol.27.7d
sol.27.7e
sol.27.7fg
sol.27.8ab
spr.27.22-32
spr.27.3a4
tur.27.4
usk.27.5a14
whb.27.1-91214
whg.27.47d
whg.27.6a
whg.27.7a
whg.27.7b-ce-k
Wcohort
R/R_0

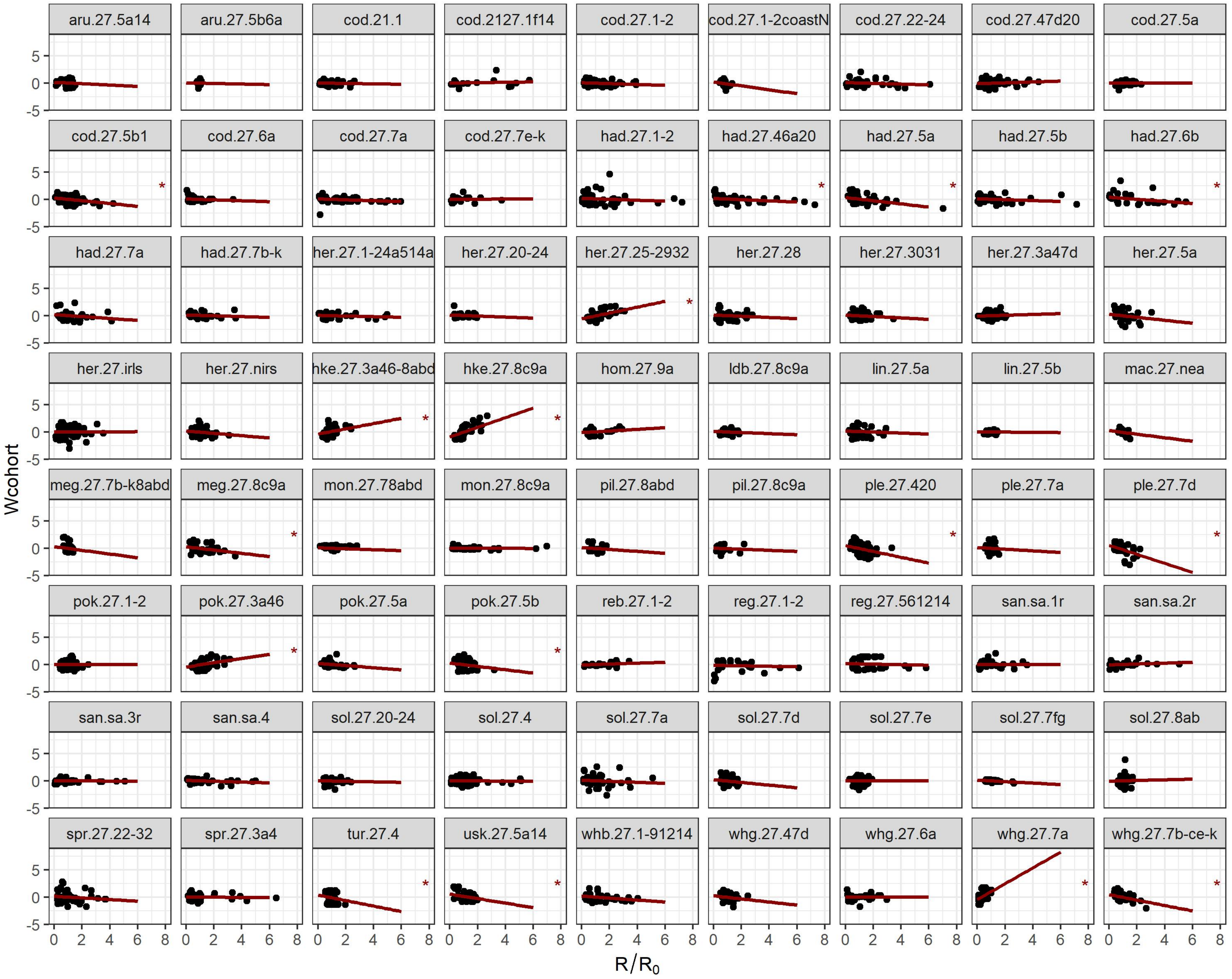
aru.27.5a14
aru.27.5b6a
cod.21.1
cod.2127.1f14
cod.27.1-2
cod.27.1-2coastN
cod.27.22-24
cod.27.47d20
cod.27.5a
cod.27.5b1
cod.27.6a
cod.27.7a
cod.27.7e-k
had.27.1-2
had.27.46a20
had.27.5a
had.27.5b
had.27.6b
had.27.7a
had.27.7b-k
her.27.1-24a514a
her.27.20-24
her.27.25-2932
her.27.28
her.27.3031
her.27.3a47d
her.27.5a
her.27.irls
her.27.nirs
hke.27.3a46-8abd
hke.27.8c9a
hom.27.9a
ldb.27.8c9a
lin.27.5a
lin.27.5b
mac.27.nea
meg.27.7b-k8abd
meg.27.8c9a
mon.27.78abd
mon.27.8c9a
pil.27.8abd
pil.27.8c9a
ple.27.420
ple.27.7a
ple.27.7d
pok.27.1-2
pok.27.3a46
pok.27.5a
pok.27.5b
reb.27.1-2
reg.27.1-2
reg.27.561214
san.sa.1r
san.sa.2r
san.sa.3r
san.sa.4
sol.27.20-24
sol.27.4
sol.27.7a
sol.27.7d
sol.27.7e
sol.27.7fg
sol.27.8ab
spr.27.22-32
spr.27.3a4
tur.27.4
usk.27.5a14
whb.27.1-91214
whg.27.47d
whg.27.6a
whg.27.7a
whg.27.7b-ce-k
Wcohort
R/R_0

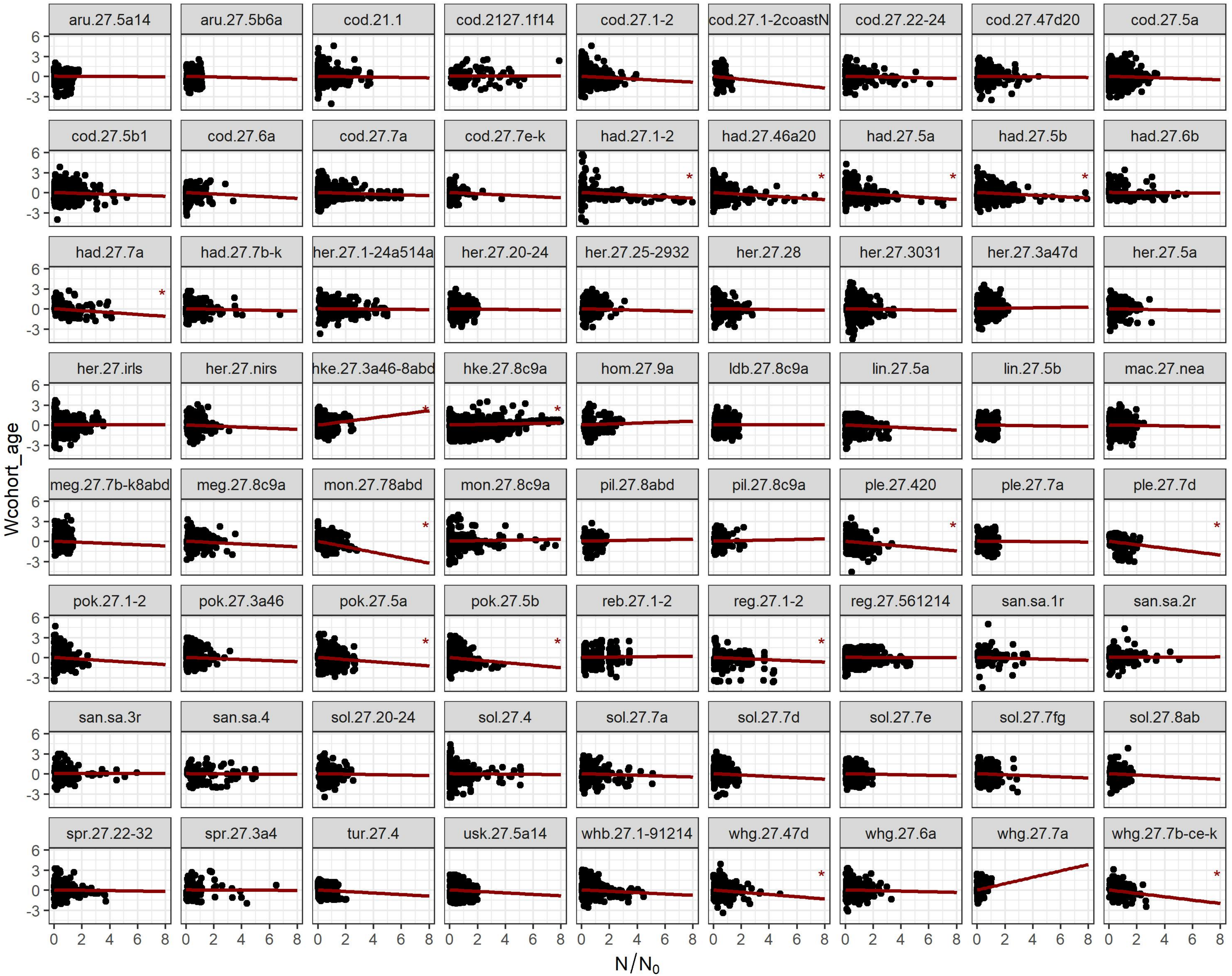

aru.27.5a14
aru.27.5b6a
cod.21.1
cod.2127.1f14
cod.27.1-2
cod.27.1-2coastN
cod.27.22-24
cod.27.47d20
cod.27.5a
cod.27.5b1
cod.27.6a
cod.27.7a
cod.27.7e-k
had.27.1-2
had.27.46a20
had.27.5a
had.27.5b
had.27.6b
had.27.7a
had.27.7b-k
her.27.1-24a514a
her.27.20-24
her.27.25-2932
her.27.28
her.27.3031
her.27.3a47d
her.27.5a
her.27.irls
her.27.nirs
hke.27.3a46-8abd
hke.27.8c9a
hom.27.9a
ldb.27.8c9a
lin.27.5a
lin.27.5b
mac.27.nea
meg.27.7b-k8abd
meg.27.8c9a
mon.27.78abd
mon.27.8c9a
pil.27.8abd
pil.27.8c9a
ple.27.420
ple.27.7a
ple.27.7d
pok.27.1-2
pok.27.3a46
pok.27.5a
pok.27.5b
reb.27.1-2
reg.27.1-2
reg.27.561214
san.sa.1r
san.sa.2r
san.sa.3r
san.sa.4
sol.27.20-24
sol.27.4
sol.27.7a
sol.27.7d
sol.27.7e
sol.27.7fg
sol.27.8ab
spr.27.22-32
spr.27.3a4
tur.27.4
usk.27.5a14
whb.27.1-91214
whg.27.47d
whg.27.6a
whg.27.7a
whg.27.7b-ce-k
Wcohort_age
N/N_0

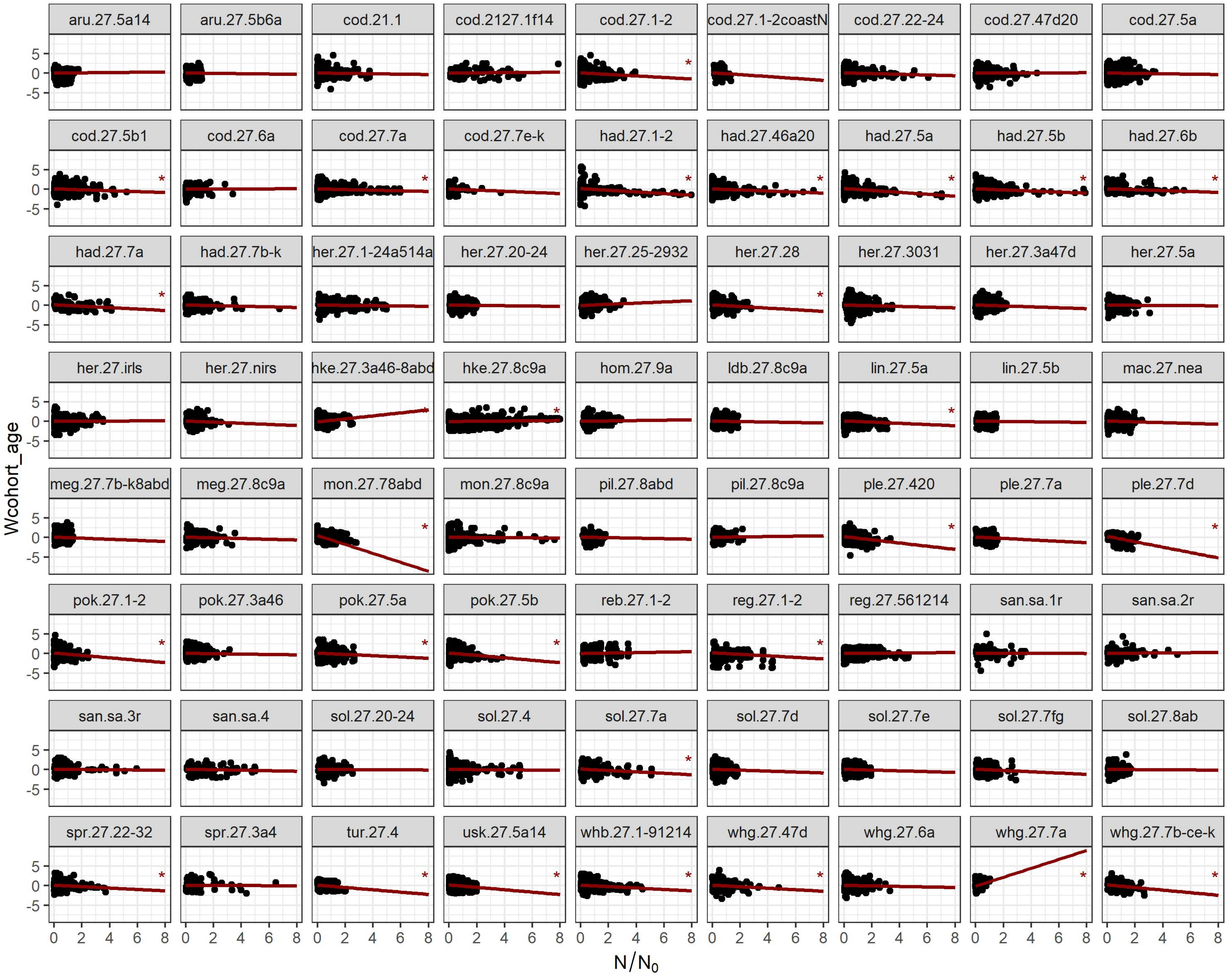
aru.27.5a14
aru.27.5b6a
cod.21.1
cod.2127.1f14
cod.27.1-2
cod.27.1-2coastN
cod.27.22-24
cod.27.47d20
cod.27.5a
cod.27.5b1
cod.27.6a
cod.27.7a
cod.27.7e-k
had.27.1-2
had.27.46a20
had.27.5a
had.27.5b
had.27.6b
had.27.7a
had.27.7b-k
her.27.1-24a514a
her.27.20-24
her.27.25-2932
her.27.28
her.27.3031
her.27.3a47d
her.27.5a
her.27.irls
her.27.nirs
hke.27.3a46-8abd
hke.27.8c9a
hom.27.9a
ldb.27.8c9a
lin.27.5a
lin.27.5b
mac.27.nea
meg.27.7b-k8abd
meg.27.8c9a
mon.27.78abd
mon.27.8c9a
pil.27.8abd
pil.27.8c9a
ple.27.420
ple.27.7a
ple.27.7d
pok.27.1-2
pok.27.3a46
pok.27.5a
pok.27.5b
reb.27.1-2
reg.27.1-2
reg.27.561214
san.sa.1r
san.sa.2r
san.sa.3r
san.sa.4
sol.27.20-24
sol.27.4
sol.27.7a
sol.27.7d
sol.27.7e
sol.27.7fg
sol.27.8ab
spr.27.22-32
spr.27.3a4
tur.27.4
usk.27.5a14
whb.27.1-91214
whg.27.47d
whg.27.6a
whg.27.7a
whg.27.7b-ce-k
Wcohort_age
N/N0

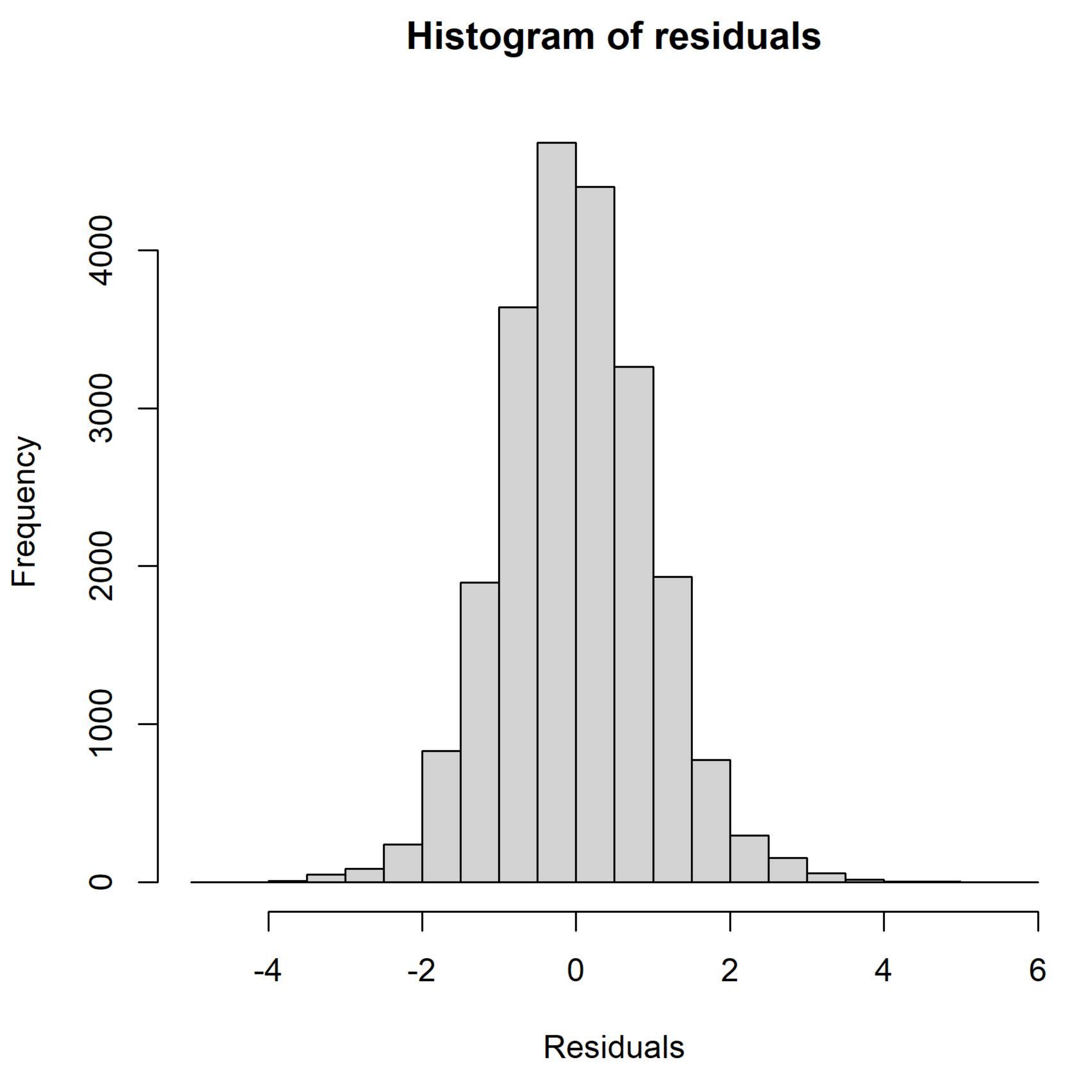
Histogram of residuals
Frequency
Residuals
0
1000
2000
3000
4000
-4
-2
0
2
4
6

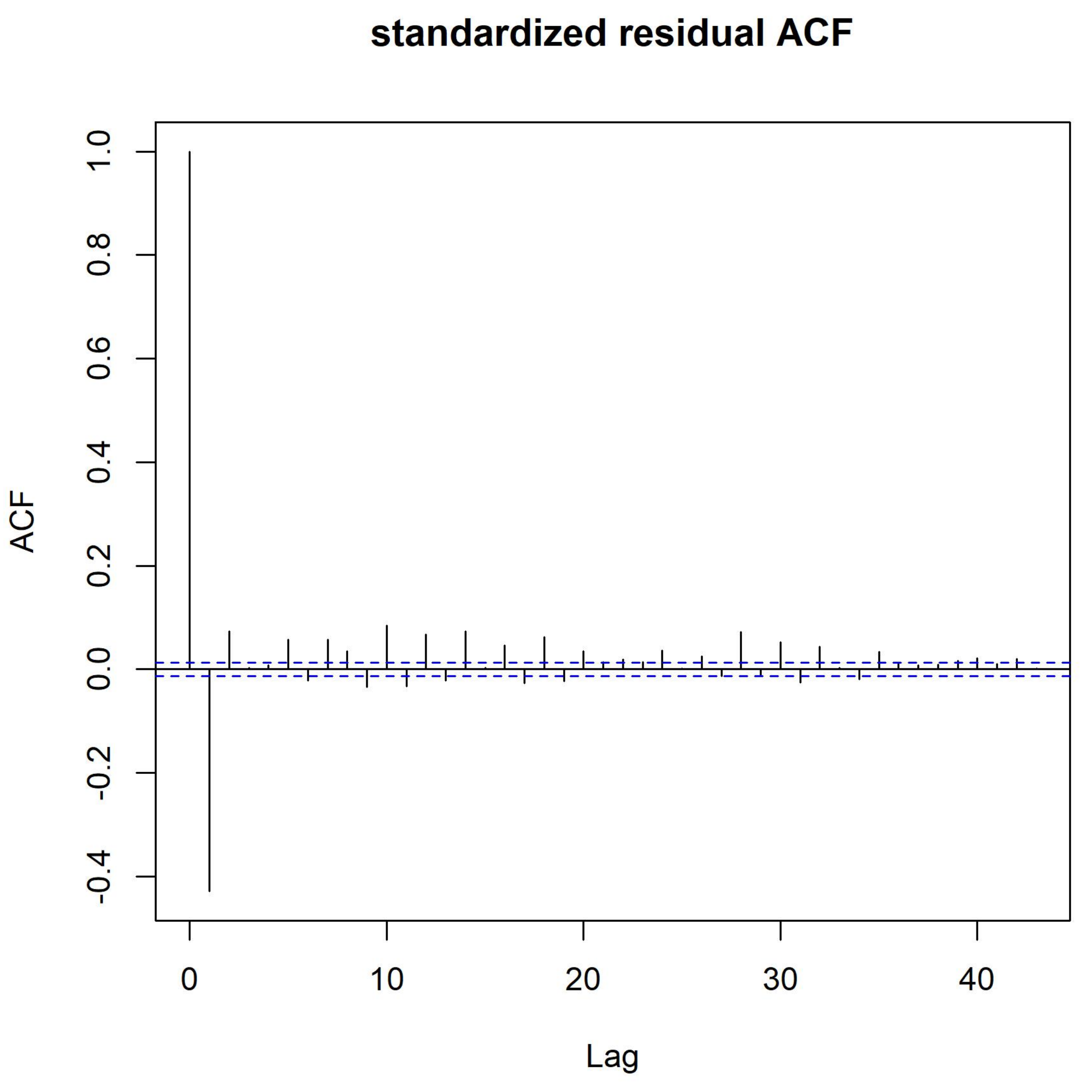
standardized residual ACF
ACF
Lag
-0.4
-0.2
0.0
0.2
0.4
0.6
0.8
1.0
0
10
20
30
40

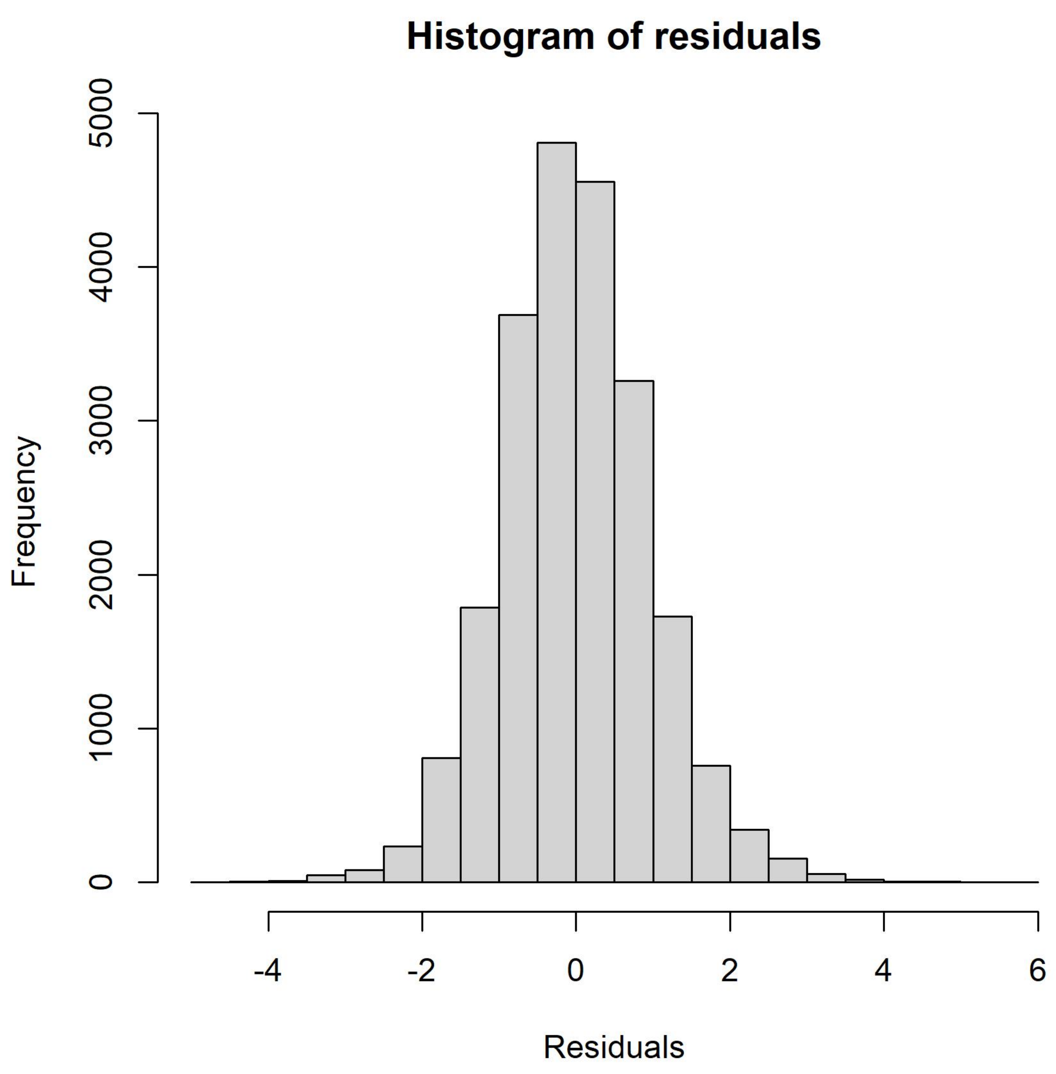
Histogram of residuals
Frequency
Residuals
0
1000
2000
3000
4000
5000
-4
-2
0
2
4
6

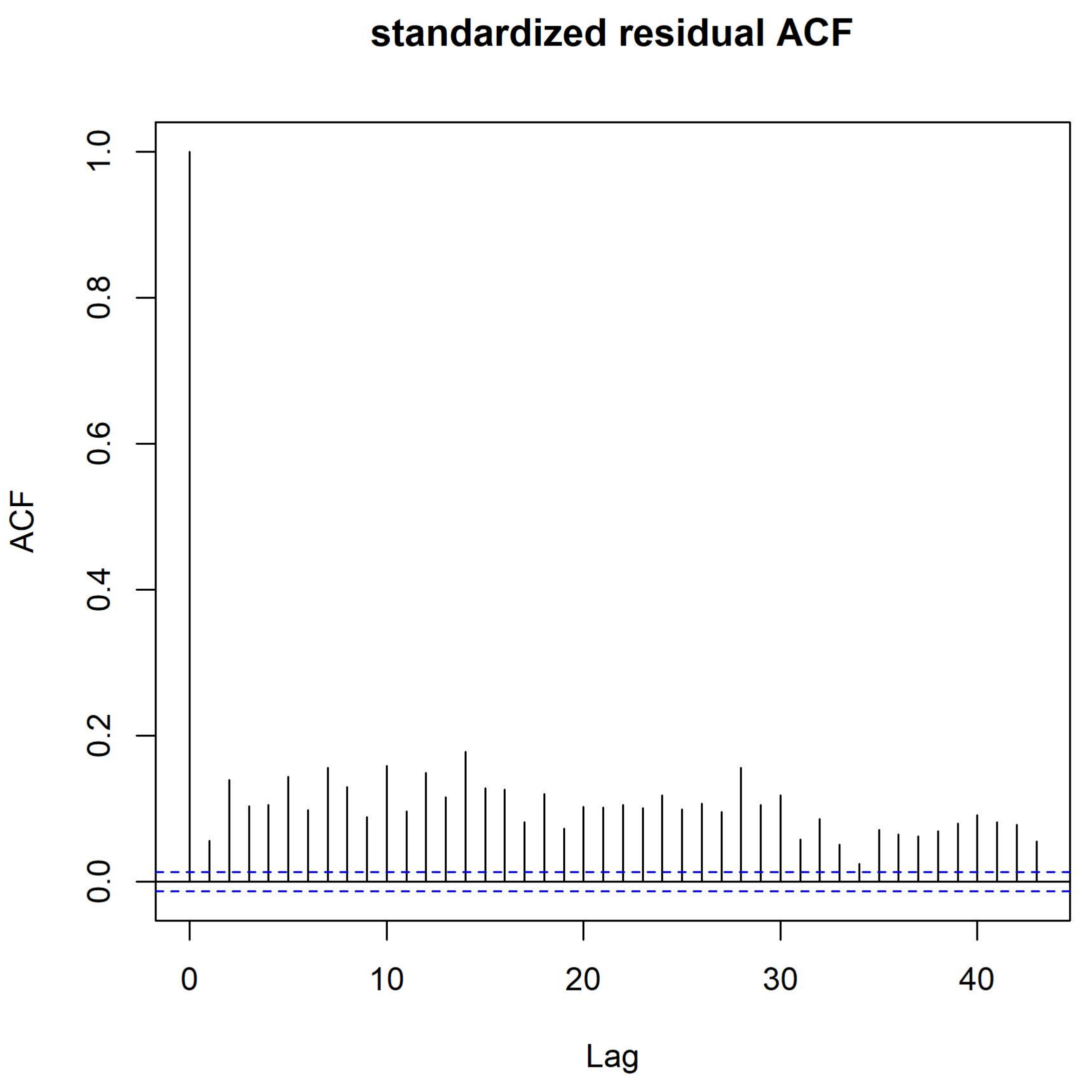
standardized residual ACF
ACF
Lag
0.0
0.2
0.4
0.6
0.8
1.0
0
10
20
30
40

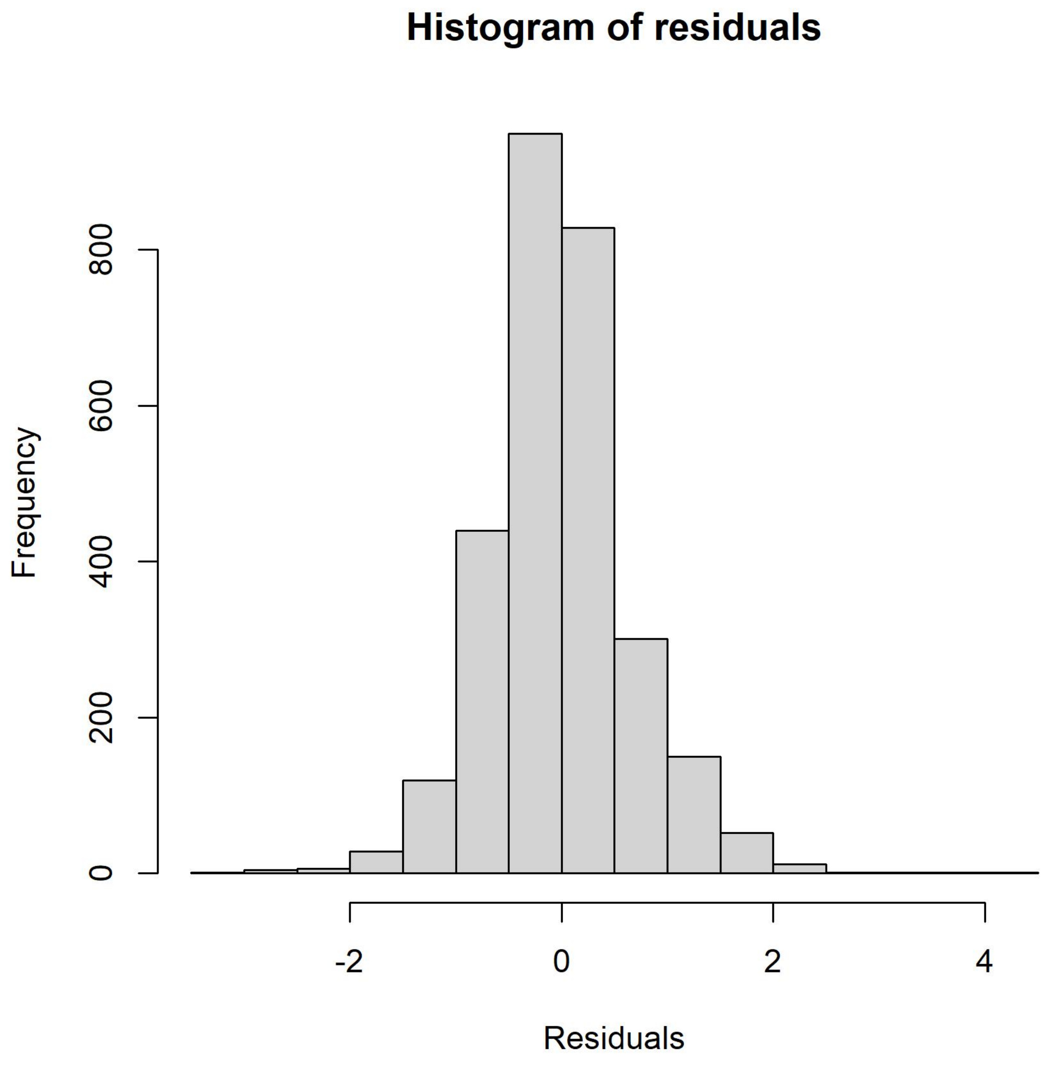
Histogram of residuals
Frequency
0
200
400
600
800
-2
0
2
4
Residuals

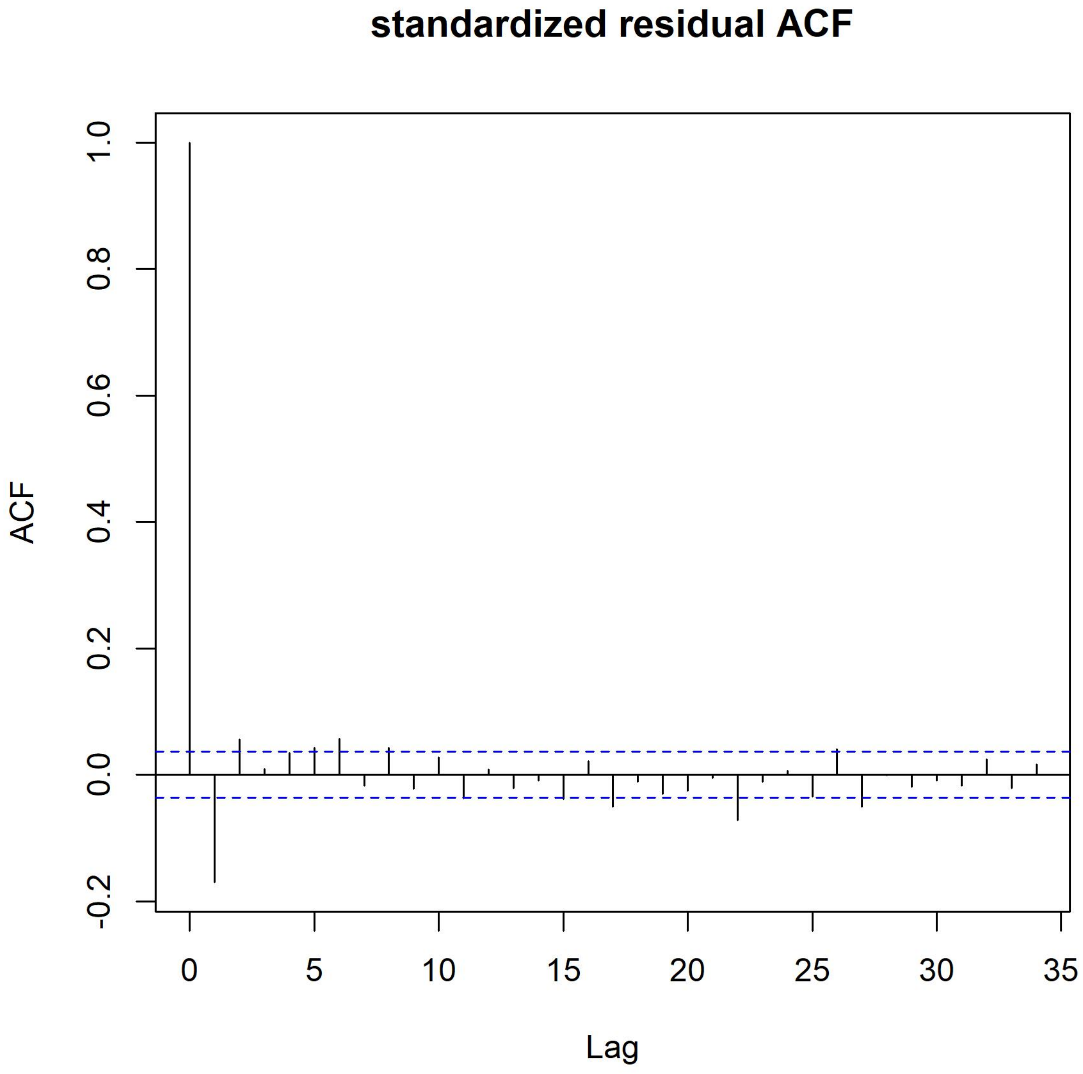
standardized residual ACF
ACF
-0.2
0.0
0.2
0.4
0.6
0.8
1.0
0
5
10
15
20
25
30
35
Lag

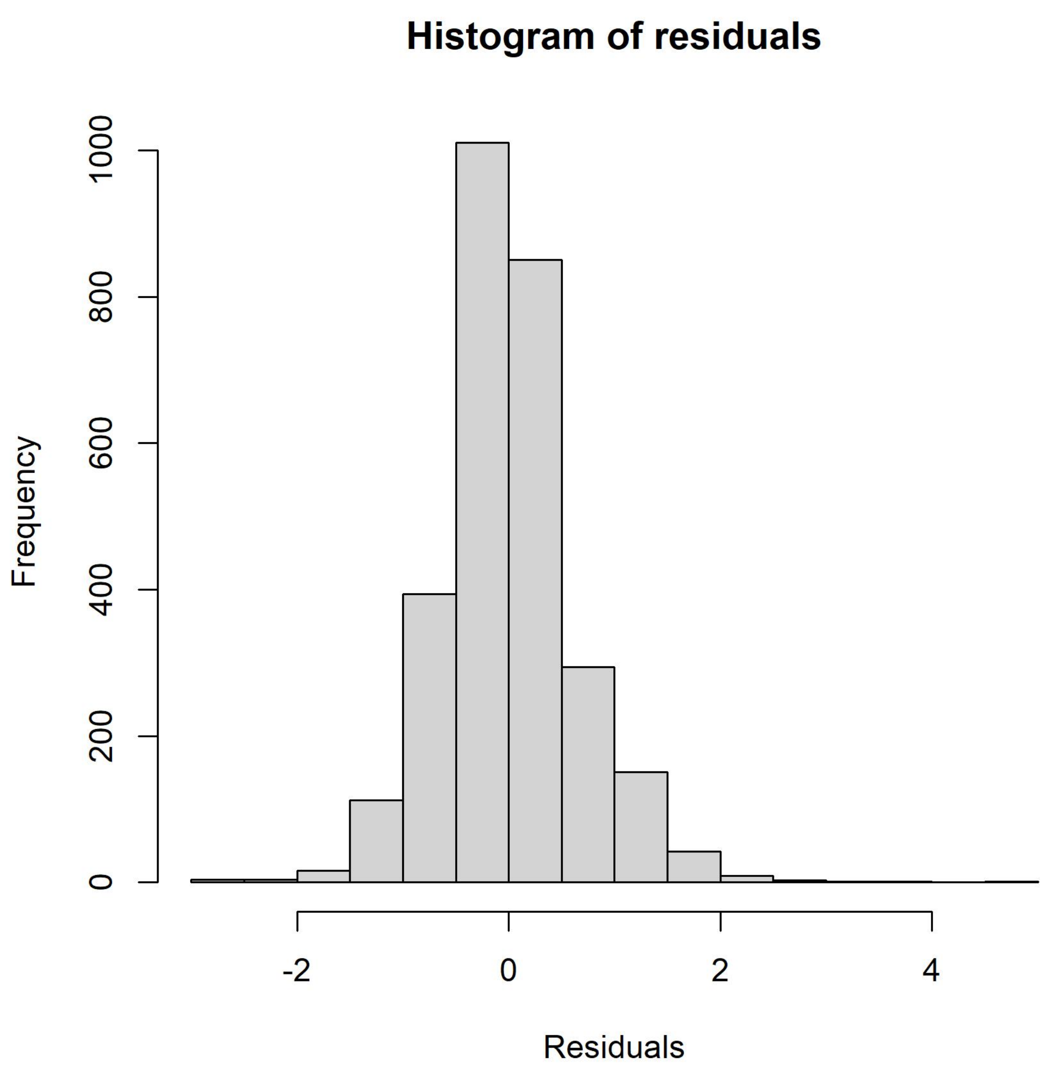
Histogram of residuals
Frequency
Residuals
0
200
400
600
800
1000
-2
0
2
4

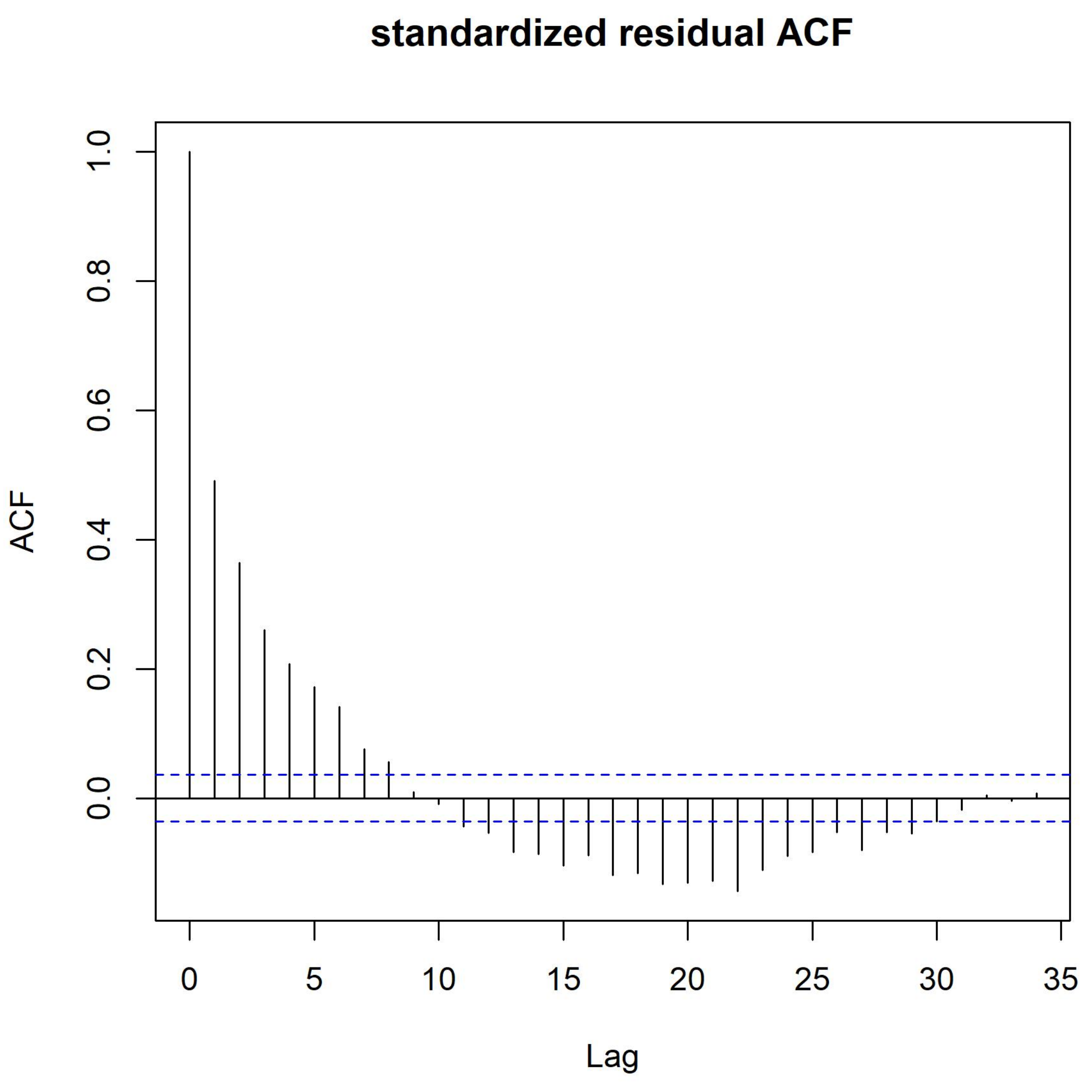
standardized residual ACF
ACF
Lag
0.0
0.2
0.4
0.6
0.8
1.0
0
5
10
15
20
25
30
35
