# Supplementary material for: Occurrence and Strength of Instantaneous and Intracohort Density‐Dependence in Northeast Atlantic Fish Stocks
Source: Ecol Evol. 2024 Oct 15;14(10):e70375. doi: 10.1002/ece3.70375 (PMC11480356; doi:10.1002/ece3.70375)
Supplement: Supplementary file 2 — Table S1. [file ECE3-14-e70375-s002.docx]

**Supplementary materials**

Table S1. List of species included in the ICES database of category 1 stocks (i.e., age structured analytical stock assessments). Stock is the ICES stock specific acronymous, species group is the grouping used in the analysis, WG is the ICES working group where the stock is assessed, Model is the assessment model used, Year is the last year of the data, B_0_ and R_0_ are the virgin biomass (in tonnes) and the associated recruitment (in 1000s individuals) as estimated by EqSim simulations. See text for more details.

Figure captions

Figure S1. Estimated relationship between the average weight of the recruits (W_recr_) and the ratio between R and R_0_ when accounting for temporal autocorrelation. The red asterisk indicated significant relationships at p<0.05.

Figure S2. Estimated relationship between the average weight of the recruits (W_recr_) and the ratio between R and R_0_ when not accounting for temporal autocorrelation. The red asterisk indicated significant relationships at p<0.05.

Figure S3. Estimated relationship between the average weight of the adults (W_old_) and the ratio between B and B_0_ when accounting for temporal autocorrelation. The red asterisk indicated significant relationships at p<0.05.

Figure S4. Estimated relationship between the average weight of the adults (W_old_) and the ratio between B and B_0_ when not accounting for temporal autocorrelation. The red asterisk indicated significant relationships at p<0.05.

Figure S5. Estimated relationship between the average weight of the population (W_std_) and the ratio between B and B_0_ when accounting for temporal autocorrelation. The red asterisk indicated significant relationships at p<0.05.

Figure S6. Estimated relationship between the average weight of the population (W_std_) and the ratio between B and B_0_ when not accounting for temporal autocorrelation. The red asterisk indicated significant relationships at p<0.05.

Figure S7. Residuals (i.e., distribution and standardized autocorrelation function) of the estimated relationship between the average weight of the recruits (W_recr_) and the ratio between R and R_0_ when accounting for temporal autocorrelation.

Figure S8. Residuals (i.e., distribution and standardized autocorrelation function) of the estimated relationship between the average weight of the adults (W_old_) and the ratio between B and B_0_ when accounting for temporal autocorrelation.

Figure S9. Residuals (i.e., distribution and standardized autocorrelation function) of the estimated relationship between the average weight of the population (W_std_) and the ratio between B and B_0_ when accounting for temporal autocorrelation.

Figure S10. Estimated relationship between the weight increment of the cohort (W_cohort_) and the ratio between R and R_0_ when accounting for temporal autocorrelation. The red asterisk indicated significant relationships at p<0.05.

Figure S11. Estimated relationship between the weight increment of the cohort (W_cohort_) and the ratio between R and R_0_ when not accounting for temporal autocorrelation. The red asterisk indicated significant relationships at p<0.05.

Figure S12. Estimated relationship between the weight increment of the cohort (W_cohort_age_) and the ratio between N and N_0_ when accounting for temporal autocorrelation. The red asterisk indicated significant relationships at p<0.05.

Figure S13. Estimated relationship between the weight increment of the cohort (W_cohort_age_) and the ratio between N and N_0_ when not accounting for temporal autocorrelation. The red asterisk indicated significant relationships at p<0.05.

Figure S14. Residuals (i.e., distribution and standardized autocorrelation function) of the estimated relationship between the weight increment of the cohort (W_cohort_) and the ratio between R and R_0_ when accounting for temporal autocorrelation.

Figure S15. Residuals (i.e., distribution and standardized autocorrelation function) of the estimated relationship between the weight increment of the cohort (W_cohort_) and the ratio between R and R_0_ when not accounting for temporal autocorrelation.

Figure S16. Residuals (i.e., distribution and standardized autocorrelation function) of the estimated relationship between the weight increment of the cohort (W_cohort_age_) and the ratio between N and N_0_ when accounting for temporal autocorrelation.

Figure S17. Residuals (i.e., distribution and standardized autocorrelation function) of the estimated relationship between the weight increment of the cohort (W_cohort_age_) and the ratio between N and N_0_ when not accounting for temporal autocorrelation.
